# Multi-parametric MRI habitat radiomics with interpretable machine learning for early prediction of axillary lymph node metastasis in triple-negative breast cancer

**DOI:** 10.3389/fmed.2026.1808030

**Published:** 2026-05-18

**Authors:** Bo Xie, Xue Peng, Yueyan Wang, Xinyuan Wen, Yindi Hu, Yihan Li, Xinnan You, Yichuan Ma

**Affiliations:** 1 The First Affiliated Hospital of Bengbu Medical University, Bengbu, China; 2 Graduate School of Bengbu Medical University, Bengbu, China; 3 West China Hospital, Sichuan University, Chengdu, China

**Keywords:** axillary lymph node metastasis, habitat radiomics, machine learning, Shapley additive explanations, triple-negative breast cancer

## Abstract

**Background:**

Accurate preoperative assessment of axillary lymph node metastasis (ALNM) in triple-negative breast cancer (TNBC) is essential for treatment planning. Conventional radiomics may overlook intratumoral heterogeneity (ITH) and lacks interpretability.

**Purpose:**

The study aimed to develop and validate an interpretable, multiparametric magnetic resonance imaging (mpMRI)-based habitat radiomics model for the preoperative prediction of ALNM in TNBC.

**Materials and methods:**

In this retrospective study, 125 patients with pathologically confirmed TNBC who underwent preoperative mpMRI were included. Tumors were manually segmented, and an unsupervised clustering approach was used to partition each lesion into intratumoral habitats. Radiomics features were extracted from both the whole tumor and habitat subregions. Clinical, conventional radiomics, habitat radiomics, and combined models were constructed using the eXtreme Gradient Boosting (XGBoost) algorithm. Model performance was evaluated using receiver operating characteristic (ROC) curve analysis, calibration curves, and decision curve analysis (DCA). Model discrimination was compared using DeLong’s test. Shapley additive explanations (SHAP) were used for model interpretation.

**Results:**

In the training set, the clinical, conventional radiomics, habitat radiomics, and combined models achieved AUCs of 0.68, 0.76, 0.79, and 0.82, respectively. In the test set, the corresponding AUCs were 0.66, 0.70, 0.74, and 0.81. The combined model showed the best performance, while the habitat radiomics model outperformed the conventional radiomics and clinical models. In the test set, SHAP analysis identified axillary lymph node (ALN) length as the most important predictor.

**Conclusion:**

The habitat radiomics model improved predictive performance over the conventional radiomics model for preoperative ALNM assessment in TNBC, and the combined model showed the highest performance. This interpretable framework highlights the value of intratumoral heterogeneity characterization for non-invasive nodal risk stratification.

## 1 Introduction

Breast cancer is the most common malignancy and the leading cause of cancer-related death among women worldwide, with a continuously increasing global burden (1, 2). Among its molecular subtypes, triple-negative breast cancer (TNBC), defined by the lack of estrogen receptor (ER), progesterone receptor (PR), and human epidermal growth factor receptor 2 (HER2) expression, accounts for approximately 10–20% of cases (3, 4). Clinically, TNBC is an aggressive breast cancer subtype associated with early recurrence, frequent distant metastasis, and limited treatment options due to the absence of actionable targets. In TNBC, nodal involvement is particularly important because it is closely linked to poor survival, increased recurrence risk, and decisions regarding neoadjuvant therapy and surgical management (5–7). Current axillary staging relies on sentinel lymph node biopsy (SLNB) or axillary lymph node dissection (ALND), both of which are invasive and associated with morbidity. With the ongoing shift toward de-escalation of axillary surgery, accurate non-invasive prediction of axillary lymph node metastasis (ALNM) has become a critical unmet clinical need (8, 9).

Biologically, TNBC is increasingly recognized as a highly intratumorally heterogeneous intratumoral heterogeneity (ITH) and evolutionarily dynamic disease, characterized by genomic instability, clonal diversification, and strong microenvironmental selection pressures that shape spatially distinct tumor ecosystems within the same lesion (10). These ecosystems consist of heterogeneous cellular populations coexisting with variable stromal, vascular, and immune components, which evolve under selective pressures such as hypoxia and nutrient gradients. This is not merely a descriptive feature but a fundamental driver of tumor progression, therapeutic resistance, and metastatic dissemination (11). Key processes driving TNBC lymphatic metastasis include epithelial–mesenchymal transition, lymphangiogenesis, chemokine-mediated trafficking, extracellular matrix remodeling, and immune evasion, which collectively facilitate tumor cell dissemination to and colonization of regional lymph nodes (12). Hypoxic and spatially ITH niches promote invasive and metastatic phenotypes, whereas microenvironmental organization can support subclonal cooperation. In TNBC, these features are closely linked to the high clinical relevance of axillary lymph node metastasis (13). Thus, a quantitative measure of intratumoral heterogeneity (ITH) could be a valuable biomarker for the prediction of ALNM.

Multiparametric magnetic resonance imaging (mpMRI) provides a non-invasive means of interrogating tumor biology by capturing complementary information on tissue microstructure, cellular density, and vascular dynamics (14). Radiomics extends this capability by extracting high-dimensional quantitative features that reflect the underlying imaging phenotype, enabling objective characterization of tumor heterogeneity (15, 16). Prior studies have demonstrated that whole-tumor radiomics can predict ALNM in TNBC (17, 18). However, these approaches rely on whole-tumor feature aggregation, which assumes spatial homogeneity, may obscure biologically aggressive subregions, and lacks model transparency (19–21). This limitation is particularly relevant in TNBC, where metastatic behavior is driven by spatially localized tumor niches rather than global tumor properties (22). Explainability analysis ensures that the results are biologically credible and clinically translatable (23).

A novel technique, referred to as habitat radiomics, was used to categorize regions with similar voxel characteristics and tumor biology into habitat subregions, thereby improving the quantification of intratumoral heterogeneity (24, 25). Habitat radiomics can preserve spatially organized microenvironments related to perfusion and cellularity and has shown promise for response prediction and nodal burden estimation in breast cancer (26–28). TreeSHAP provides exact Shapley value solutions for tree-based models with polynomial time complexity, enabling both accurate and computationally efficient interpretability (29).

Therefore, the study aimed to develop and validate an interpretable machine learning model integrating mpMRI habitat radiomics, conventional whole-tumor radiomics, and clinicopathological factors for the preoperative prediction of ALNM in TNBC. We further applied SHAP to quantify feature contributions and improve interpretability.

## 2 Materials and methods

### 2.1 Patient population

This retrospective study was approved by the ethics committee of the First Affiliated Hospital of Bengbu Medical University. (No. 115 [2025]). Furthermore, the study was exempt from the requirement for informed consent from the participants.

**Figure 1:**
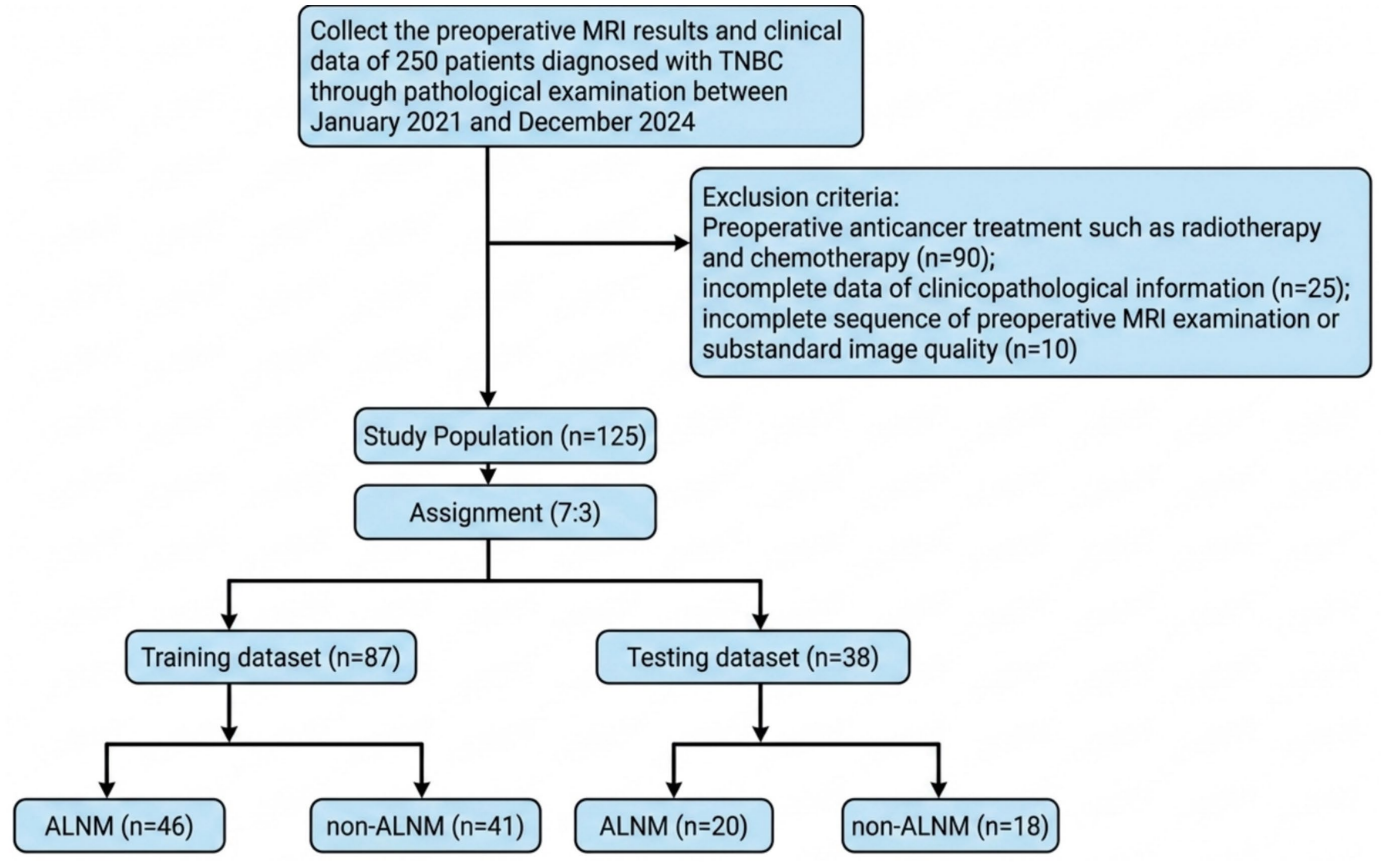
Flowchart of patient inclusion and exclusion criteria for the study.

Consecutive patients with histopathologically confirmed TNBC who underwent preoperative breast MRI from January 2021 to December 2024 were screened. The inclusion criteria were as follows: (1) pathologically confirmed TNBC; (2) preoperative mpMRI, including T2WI, DWI, and DCE-MRI, performed within 2 weeks before surgery; and (3) definitive axillary lymph node (ALN) status confirmed by postoperative pathology following ALND. The exclusion criteria were as follows: (1) receipt of antitumor therapy prior to MRI, (2) incomplete clinicopathological data, and (3) missing sequences or non-diagnostic image quality. A total of 125 patients were randomly divided into training and testing datasets, with 87 patients (70%) used for training the prediction model and 38 patients (30%) used as an independent testing dataset. Given the relatively balanced distribution of ALNM and non-ALNM cases (66 vs. 59), comparable class proportions were maintained after random splitting. The inclusion and exclusion processes and the grouping results are shown in Figure 1. Baseline characteristics of the study population are summarized in Table 1.

**Table 1:** Characteristics of 125 TNBC patients in different datasets.

Feature	Total (*n* = 125)	Training set (*n* = 87)	Test set (*n* = 38)	*P-*value	Non-ALNM (*n* = 59)	ALNM (*n* = 66)	*P-*value
Age, mean (SD)	49.1(9.6)	49.6(9.5)	47.7(9.7)	0.313	47.4 (9.9)	50.5 (9.2)	0.076
Tumor length, mean (SD)	28.6(14.5)	30.4(16.4)	24.4(7.3)	0.005	26.6 (9.6)	30.4 (17.7)	0.150
ALN length, mean (SD)	12.1(11.6)	12.9(12.2)	10.2(10.1)	0.209	5.0 (5.9)	18.4 (11.9)	<0.001
Alb/Glb mean (SD)	1.6(0.3)	1.7(0.3)	1.6(0.3)	0.093	1.7 (0.3)	1.6 (0.3)	0.361
TC, mean (SD)	4.6(0.9)	4.6(0.9)	4.7(0.9)	0.433	4.4 (0.8)	4.8 (1.0)	0.021
HDL-C, mean (SD)	1.3(0.3)	1.2(0.3)	1.3(0.3)	0.204	1.3 (0.3)	1.3 (0.3)	0.478
LDL-C, mean (SD)	2.7(0.7)	2.7(0.7)	2.7(0.9)	0.580	2.5 (0.7)	2.8 (0.7)	0.023
TIC, *N* (%):				0.642			<0.001
Inflow or platform curve	50 (40.0%)	33 (37.9%)	17 (44.7%)		34 (57.6)	16 (24.2)	
Washout curve	75 (60.0%)	54 (62.1%)	21 (55.3%)		25 (42.4)	50 (75.8)	
BI_RADS, *N* (%):				0.307			0.624
4a4b	17 (13.6%)	11 (12.6%)	6 (15.8%)		12 (20.3)	5 (7.6)	
4c	33 (26.4%)	20 (23.0%)	13(34.2%)		21 (35.6)	12 (18.2)	
5	75 (60.0%)	56 (64.4%)	19 (50.0%)		26 (44.1)	49 (74.2)	
Grade, *N* (%):				0.151			<0.001
1–2	49 (39.2%)	30 (34.5%)	19 (50.0%)		35 (59.3)	14 (21.2)	
3	76 (60.8%)	57 (65.5%)	19 (50.0%)		24 (40.7)	52 (78.8)	
Ki-67, *N* (%):				0.849			0.05
<20%	36 (28.8%)	26 (29.9%)	10 (26.3%)		22 (37.3)	14 (21.2)	
≥20%	89 (71.2%)	71 (70.1%)	28 (73.7%)		37 (62.7)	52 (78.8)	
Menopausal status, *N* (%):				0.135			0.494
Negative	68 (54.4%)	43 (49.4%)	25 (65.8%)		34 (57.6)	4 (51.5)	
Positive	57 (45.6%)	44 (50.6%)	13 (34.2%)		25 (42.4)	32 (48.5)	

### 2.2 Clinicopathological data collection

Clinicopathological data were retrospectively retrieved from institutional medical records and pathology reports. Variables included age, menopausal status, maximum tumor diameter, and axillary lymph node (ALN) diameter on MRI; serum albumin-to-globulin ratio (Alb/Glb); total cholesterol (TC); high-density lipoprotein cholesterol (HDL-C); and low-density lipoprotein cholesterol (LDL-C). The Ki-67 index was assessed by immunohistochemistry. Ki-67 expression was dichotomized using a 20% cutoff according to the St. Gallen consensus (30). HER2 negativity was confirmed by immunohistochemistry and/or fluorescence *in situ* hybridization (31). Axillary lymph node status was evaluated based on the NCCN and ASCO guidelines (9).

### 2.3 Image acquisition

All patients underwent preoperative MRI using a 3.0 Tesla Philips (Netherlands) Achieva dual-gradient superconducting system. Detailed scanning parameters are provided in Supplementary material S1.

### 2.4 Image preprocessing

To ensure reproducibility and comparability of radiomics features, all patients’ MRI data underwent standardized image preprocessing. First, all sequences were spatially co-registered to the DCE sequence, which served as the reference, using deformable registration (symmetric normalization, SyN) implemented in Advanced Normalization Tools (ANTs, version 1.8.1). Second, to account for differences in voxel spacing across patients and imaging acquisitions, all images were resampled to an isotropic voxel size of 1 × 1 × 1 mm^3^ using linear interpolation. The corresponding region-of-interest (ROI) masks were resampled using nearest-neighbor interpolation to preserve label integrity. Third, to mitigate the influence of extreme intensity values, voxel intensities within each tumor VOI were subjected to percentile-based clipping. Finally, intensity normalization was performed on a per-patient, per-sequence basis using *Z*-score standardization. This normalization facilitated the comparability of radiomics features across patients and imaging sequences. The workflow is shown in Supplementary material S2.

### 2.5 Tumor segmentation

A primary reviewer (Radiologist A, with 20 years of experience in breast MRI diagnosis) independently delineated the ROIs on DCE-MRI (the third contrast phase) for all patients using ITK-SNAP software (version 3.8.0). The ROIs were then transferred to the co-registered T2WI and DWI sequences to ensure consistent tumor regions across all modalities. These ROIs were used as the final segmentation for subsequent radiomics analysis. Another reviewer (Radiologist B, with 10 years of experience in breast MRI diagnosis) delineated ROIs using the same method to assess the consistency of radiomics features and interobserver variability.

### 2.6 Habitat generation

Tumor subregion segmentation was performed using an unsupervised habitat imaging framework to characterize intratumoral heterogeneity. To ensure both the mathematical robustness of the radiomics features and the preservation of biological tumor boundaries, we employed a rigorous “extract-then-pool” framework rather than computing features directly within the supervoxels. First, radiomics features were continuously extracted at the voxel level across the entire tumor region of interest (ROI) using a fixed-size sliding window approach. Subsequently, these localized feature values were spatially aggregated (via mean pooling) within the precise boundaries of the independently generated SLIC supervoxels. K-means clustering was then applied to cluster supervoxels with similar radiomics features into distinct subregions, referred to as habitats. K-means clustering was performed with the number of clusters ranging from 2 to 10. The optimal number of clusters was determined based on the silhouette coefficient. The habitat generation pipeline is depicted in Supplementary material S3. All analyses were implemented in *Python* (version 3.12.10).

### 2.7 Feature extraction

Features were extracted from the original whole tumor and habitats using the open-source software *Python*, which extensively employs the *PyRadiomics* library for feature extraction. In this study, handcrafted features were extracted and classified into three categories: (I) shape features, (II) first-order features, and (III) texture features, including gray-level co-occurrence matrix (GLCM), gray-level dependence matrix (GLDM), gray-level run length matrix (GLRLM), gray-level size zone matrix (GLSZM), and neighborhood gray-tone difference matrix (NGTDM). During feature extraction, wavelet transforms (with eight decomposition levels) and Laplacian of Gaussian (LoG) filtering (with Gaussian kernel *σ* values of 3.0 and 4.0) were applied. These operations enhance image information across different scales and frequencies, thereby facilitating the extraction of texture features with superior discriminative power. Features from habitat subregion 1 were labeled T2WI_habitat1, DWI_ habitat1, and DCE (dyn)_ habitat 1, following a similar naming convention for subsequent regions. Conventional radiomics features were named after the sequence name. Details of feature extraction are provided in Supplementary material S4.

### 2.8 Feature selection

For interobserver reproducibility analysis, two radiologists independently segmented the tumor on DCE-MRI for all patients. Using each segmentation result, the complete feature extraction pipeline was repeated, including the extraction of whole-tumor radiomics features and habitat-based subregional radiomics features. The intraclass correlation coefficient (ICC) was calculated for each feature to assess robustness to segmentation variability. An ICC threshold of 0.90 was adopted to ensure excellent reproducibility, as values above 0.90 are widely considered to indicate high interobserver agreement and feature robustness. Subsequently, feature selection was performed separately for the whole-tumor feature pool, the habitat subregion feature pool, and the unified feature pool. The *Z*-score function was used to standardize the three pools, eliminating the influence of units and dimensions among features and preventing features with larger values from dominating the selection process. A *t-*test or the Mann–Whitney *U* test was used, and only features with a *p*-value of < 0.05 were retained. Spearman’s correlation coefficient analysis (cutoff = 0.90) was carried out. For any pair of features with a correlation coefficient greater than 0.9, one feature was retained, while the other was removed. The minimum redundancy–maximum relevance (mRMR) method was used to eliminate redundant and irrelevant features. The least absolute shrinkage and selection operator (LASSO) algorithm was used to avoid overfitting. Through adjustment and 10-fold cross-validation, the optimal features were selected. All feature selection steps were performed only in the training set. Details of feature selection are provided in Supplementary material S5.

### 2.9 Model construction

In the training set, univariable logistic regression was used to evaluate clinicopathological variables associated with ALNM status, and variables with a *p*-value of < 0.05 were entered into multivariable logistic regression. Clinicopathological variables identified as independent predictors in the multivariable logistic regression analysis were utilized to develop the clinical model. The selected whole-tumor radiomics features were used to construct the conventional radiomics model, while habitat radiomics features were employed to establish the habitat model. In addition, a combined model integrating significant clinicopathological variables, whole-tumor radiomics features, and habitat radiomics features was developed. The model development process was supervised, and the eXtreme Gradient Boosting (XGBoost) algorithm was employed.

The XGBoost algorithm was utilized to develop four predictive models for ALNM in the training cohort. The hyperparameters of the XGBoost model were optimized using 5-fold stratified cross-validation within the training set. The training set was partitioned into five subsets; in each iteration, four subsets were used for training and the remaining subset for validation. This process was repeated five times so that each subset served as a validation fold once. Hyperparameter tuning was performed within each training fold using grid search combined with cross-validation, where the mean AUC across the validation folds was used as the criterion for selecting the optimal hyperparameter combination. The final model was configured using the optimal hyperparameters identified during cross-validation, refitted on the entire training cohort, and subsequently evaluated on the test set. Details of model construction and verification are provided in Supplementary material S6. The working principle of XGBoost is provided in Supplementary material S7.

### 2.10 Model evaluation and interpretation

**Figure 2:**
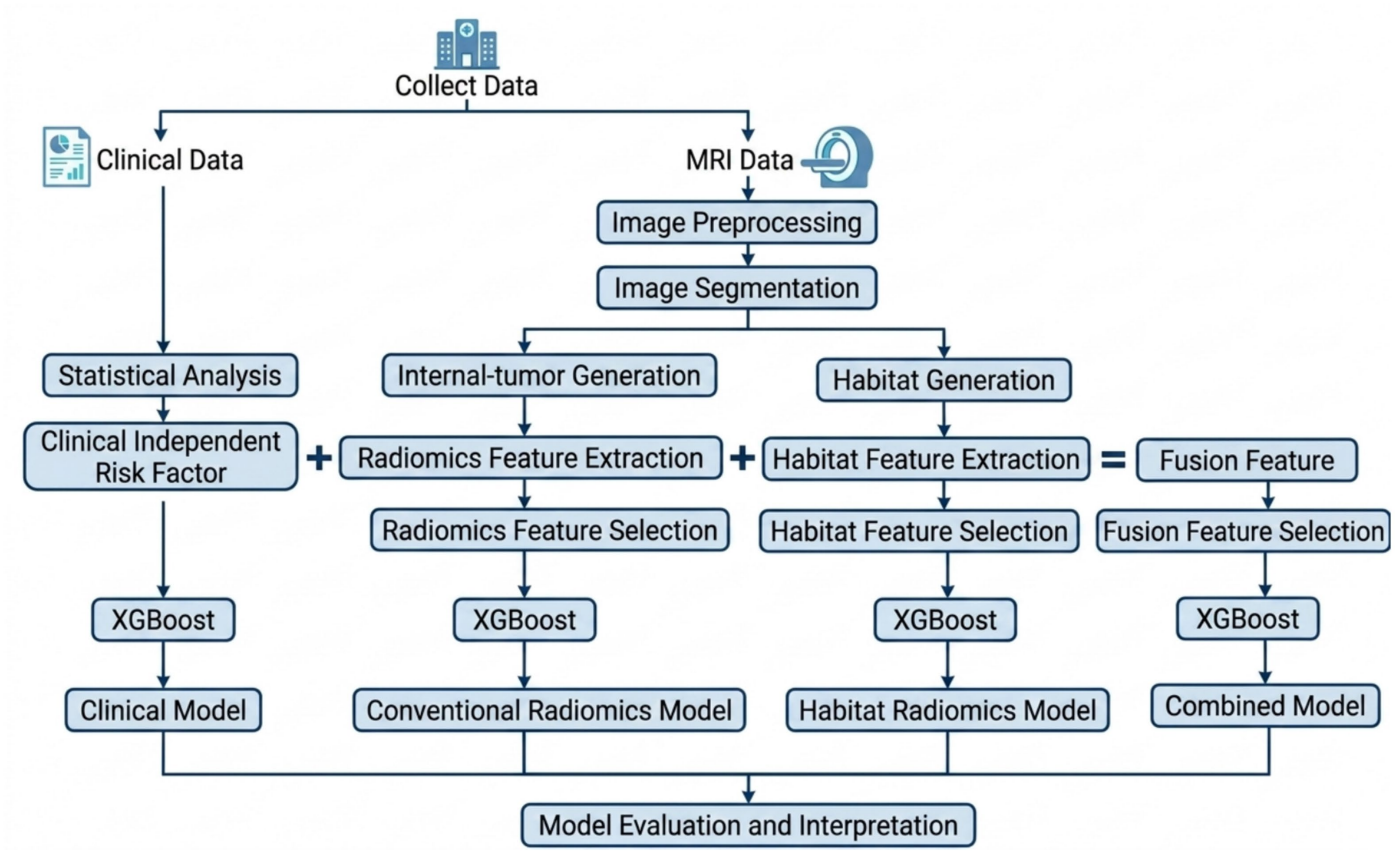
A simplified radiomics workflow for intratumoral radiomics and habitat analysis, incorporating multiple predictive models.

Model discrimination was assessed in the test set using receiver operating characteristic (ROC) curve analysis, with calculation of the AUC. Differences in AUCs between the models were compared using DeLong’s test in the test set. A *p*-value of less than 0.05 was considered statistically significant. Model calibration was assessed using calibration curves and the Hosmer–Lemeshow goodness-of-fit test. The Hosmer–Lemeshow test evaluates the agreement between predicted probabilities and observed outcomes. A non-significant result (*p* > 0.05) indicates good calibration of the model. Decision curve analysis (DCA) was performed to evaluate the clinical utility of the models by quantifying the net benefit across a range of threshold probabilities. To quantify diagnostic performance, sensitivity, specificity, accuracy, precision, positive predictive value (PPV), and negative predictive value (NPV) were calculated from confusion matrices generated using an optimal classification threshold of 0.5. Details of model evaluation are provided in Supplementary material S8. Model interpretation was facilitated through SHapley Additive exPlanations (SHAP) to visualize the impact of conventional radiomics features, habitat radiomics features, and clinicopathological variables on the prediction of the combined model. Details of SHAP analysis are provided in Supplementary material S9. The whole workflow of this study is shown in Figure 2.

### 2.11 Statistical analysis

Statistical analyses were conducted using IBM *SPSS* Statistics (version 25.0) and *R* statistical software (version 4.1.2). The normality of continuous variables was assessed using the Shapiro–Wilk test. Continuous variables, shown as mean (standard deviation), were compared between the ALNM and non-ALNM groups using a *t-*test or the Mann–Whitney *U* non-parametric test according to data normality. Categorical variables were analyzed using either chi-squared tests or Fisher’s exact tests and were expressed as proportions.

## 3 Results

### 3.1 Patient characteristics

As shown in Tables 1, a total of 125 TNBC patients were included (66 with ALNM and 59 with non-ALNM), with comparable baseline characteristics between the training and test sets (all *p* > 0.05). In the training set, univariable logistic regression analysis identified axillary lymph node length, TIC type (washout curve), Nottingham grade G3 (poorly differentiated / high grade), BI-RADS-5, and Ki-67 as independent risk factors for ALNM (all *p* < 0.05). Multivariable analysis identified lymph node size, washout TIC pattern, and Nottingham grade G3 as independent predictors (all *p* < 0.05). Overall, ALNM was primarily associated with nodal morphology, vascular characteristics, and tumor grade. Detailed results of the univariable and multivariable analyses are presented in Table 2.

**Table 2:** Univariable and multivariable logistic regression analyses in the training set.

Feature	Non-ALNM (*n* = 41)	ALNM (*n* = 46)	Univariable analysis OR (95%CI)	*P*-value	Multivariable analysis OR (95%CI)	*P*-value
Age	48.206 ± 11.467	50.038 ± 9.761	1.017(0.976–1.061)	0.423		
Tumor length	24.118 ± 7.95	29.925 ± 18.461	1.04(0.994–1.088)	0.0929		
ALN length	4.471 ± 6.076	18.34 ± 12.332	1.183(1.099–1.273)	<0.001	1.208 (1.091–1.337)	0.0003
Alb/Glb	1.696 ± 0.294	1.645 ± 0.31	0.577(0.139–2.4)	0.4494		
TC	4.533 ± 0.877	4.767 ± 0.966	1.32 (0.818–2.128)	0.2553		
HDL-C	1.259 ± 0.279	1.274 ± 0.3	1.201 (0.269–5.364)	0.8107		
LDL-C	2.642 ± 0.798	2.797 ± 0.723	1.329 (0.735–2.402)	0.3465		
Inflow or platform curve	17 (50.0%)	10 (18.868%)				
Washout curve	17 (50.0%)	43 (81.132%)	4.3 (1.643–11.253)	0.003	4.625 (1.162–18.398)	0.0297
BI-RADS 4a-4b	8 (23.529%)	4 (7.547%)				
BI-RADS 4c	13 (38.235%)	11 (20.755%)	1.692 (0.399–7.172)	0.4752	1.219 (0.192–7.749)	0.8338
BI-RADS 5	13 (38.235%)	38 (71.698%)	5.847 (1.508–22.676)	0.0107	0.836 (0.135–5.153)	0.8465
Grade 1–2	22 (64.706%)	13 (24.528%)				
Grade 3	12 (35.294%)	40 (75.472%)	5.641 (2.2–14.461)	0.0003	6.916 (1.756–27.242)	0.0057
Ki-67 < 20%	15 (44.118%)	10 (18.868%)				
Ki-67 ≥ 20%	19 (55.882%)	43 (81.132%)	3.395 (1.293–8.913)	0.0131	1.869 (0.439–7.956)	0.3975
Menopause (−)	21 (61.765%)	28 (52.83%)				
Menopause (+)	13 (38.235%)	25 (47.17%)	1.442 (0.6–3.467)	0.4131		

### 3.2 Feature extraction and selection

Based on silhouette coefficient analysis, *K* = 3 was identified as the optimal number of clusters, and three distinct tumor habitats were therefore used for subsequent analysis. In total, 1,037 types of radiomics features were extracted from the whole-tumor region and each specific habitat. A total of 3,111 (1,037 × 3 sequences) conventional whole-tumor radiomics features, 9,333 (1,037 × 3 sequences × 3 habitats) habitat radiomics features, and 12,444 fusion features were extracted for further selection. Following feature screening and dimensionality reduction, 12 conventional radiomics features and 10 habitat radiomics features were utilized to construct the conventional radiomics model and the habitat radiomics model, respectively. The combined model integrated 31 features, including 18 conventional radiomics features, 10 habitat radiomics features, and three clinical features. The dimensionality reduction path and the optimal features are shown in Supplementary material S5.

### 3.3 Model performance and comparison

**Figure 3:**
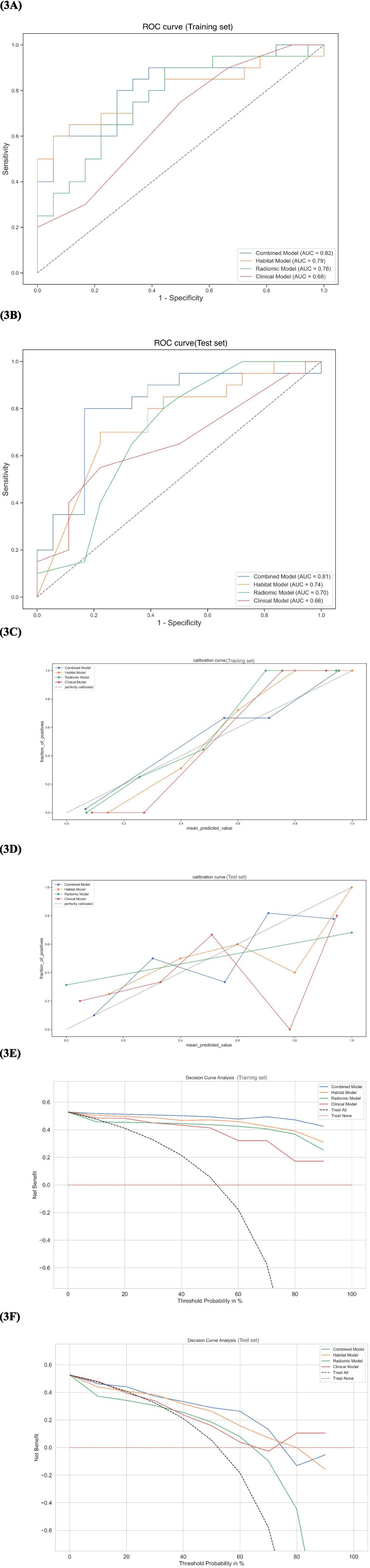
Prediction model validation. **(A,B)** Comparison of ROC curves for the four models. **(C,D)** Calibration curves of the four models. **(E,F)** Comparative DCA of the four models.

The ROC curves of the four models in the training and test sets are shown in Figures 3A,B. The clinical model achieved an AUC of 0.68 (95%CI: 0.56–0.79) in the training set and 0.66 (95%CI: 0.48–0.83) in the test set. The conventional radiomics model achieved an AUC of 0.76 (95%CI: 0.65–0.86) in the training set and 0.70 (95%CI: 0.53–0.86) in the test set. The habitat radiomics model achieved an AUC of 0.79 (95% CI: 0.69–0.88) in the training set and 0.74 (95% CI: 0.57–0.90) in the testing set. The combined model achieved the highest predictive performance, with an AUC of 0.82 (95% CI: 0.73–0.90) in the training set and 0.81 (95% CI: 0.66–0.95) in the test set. Pairwise comparisons between the three AUCs in the test set were performed using DeLong’s test. Calibration curves demonstrated good agreement between predicted and observed probabilities, which was further supported by the Hosmer–Lemeshow test (*p* > 0.05) (Figures 3C,D). Decision curve analysis (DCA) demonstrated that the combined model offered the greatest net benefit across a broad range of threshold probabilities (Figures 3E,F). The AUC, accuracy, sensitivity, specificity, precision, PPV, and NPV of each model are shown in Table 3. Detailed model performance results and comparisons are shown in Supplementary material S8.

**Table 3:** Model performance in the training and test sets.

**Model**	**Cohort**	**AUC**	**95%CI**	**Accuracy**	**Sensitivity**	**Specificity**	**Precision**	**PPV**	**NPV**
Combined model	Training set	0.82	0.73–0.90	0.977	0.978	0.976	0.978	0.929	0.976
	Test set	0.81	0.66–0.95	0.837	0.850	0.861	0.808	0.861	0.786
Habitat radiomics model	Training set	0.79	0.69–0.88	0.943	0.913	0.976	0.977	0.912	0.909
	Test set	0.74	0.57–0.90	0.726	0.750	0.722	0.750	0.736	0.722
Conventional radiomics model	Training set	0.76	0.65–0.86	0.908	0.870	0.951	0.952	0.906	0.867
	Test set	0.70	0.53–0.86	0.708	0.707	0.656	0.652	0.600	0.667
Clinical model	Training set	0.68	0.56–0.79	0.885	0.891	0.878	0.891	0.832	0.878
	Test set	0.66	0.48–0.83	0.632	0.650	0.625	0.658	0.602	0.643

### 3.4 Model interpretability

**Figure 4:**
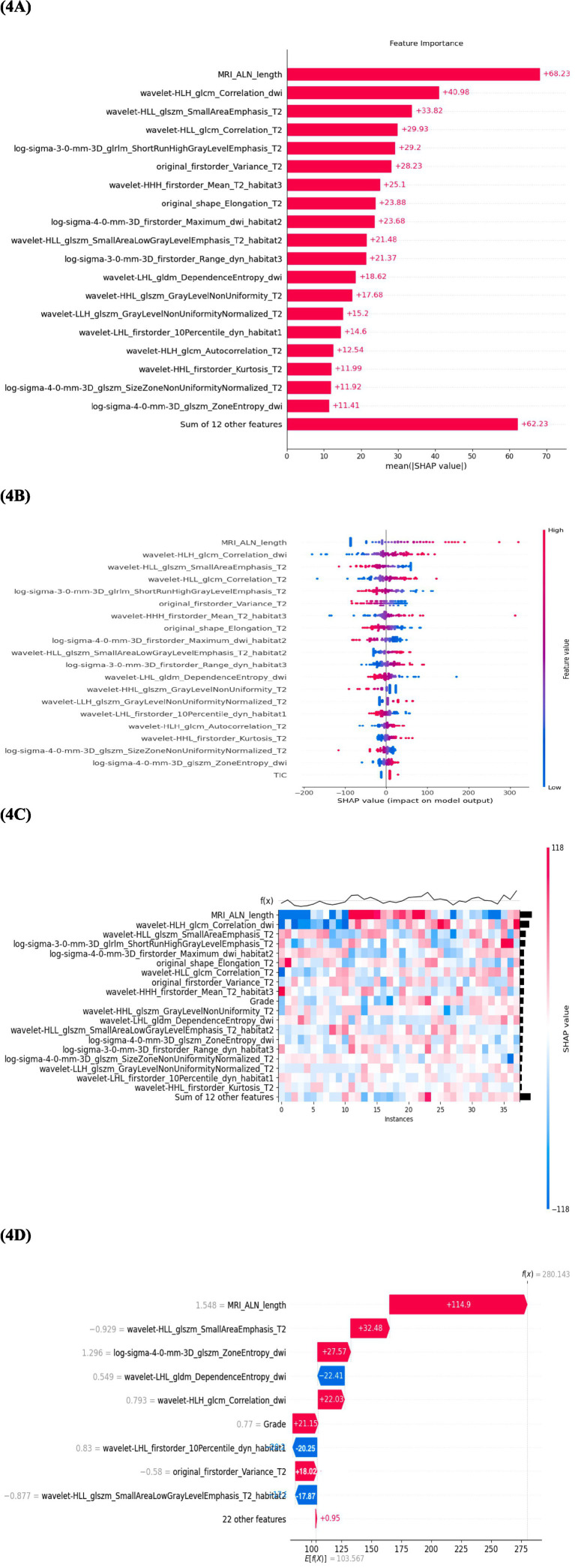
Interpretability analysis of the radiomics model using SHAP values. **(A)** SHAP feature importance bar plot: Features are ranked by their overall importance, quantified as the mean absolute SHAP value across all samples in the test set. **(B)** SHAP beeswarm plot: Each dot represents an individual sample, with color representing the feature value (red for high values and blue for low values). **(C)** SHAP heatmap: The horizontal axis represents individual patient samples from the test set, and the vertical axis lists radiomic features ranked by importance. **(D)** SHAP waterfall plot for a representative case: The plot illustrates the cumulative contribution of individual features to the final model output for a single sample.

Shapley additive explanations analysis was used to assess the contribution of individual features in the combined model (Figure 4). The model integrated clinical and morphological variables, conventional radiomics features, and habitat-based radiomics features. In the global importance ranking, *MRI_ALN_length* showed the highest individual SHAP value, while several radiomics features derived from T2WI, DWI, and DCE-MRI were also among the most influential predictors (Figure 4A). Notably, multiple top-ranked features were derived from predefined intratumoral habitats, indicating that subregional imaging information contributed substantially to model prediction. The SHAP beeswarm plot showed that ALNM prediction was influenced by the combined effects of the three feature categories, although both the magnitude and direction of feature contributions varied across patients (Figure 4B). Similar inter-patient heterogeneity was observed in the heatmap (Figure 4C). In the representative waterfall plot, the final prediction resulted from the cumulative contribution of clinical, conventional radiomic, and habitat-based radiomics features rather than from a single variable alone (Figure 4D).

## 4 Discussion

In this study, we performed habitat radiomics analysis of breast MRI to develop a model for the early prediction of tumor ALNM. The habitat radiomics model (AUC = 0.74) outperformed both the clinical and conventional radiomics models in the test set. Integration of habitat radiomics features with conventional radiomics features and clinical factors into a combined model (AUC = 0.81) further enhanced predictive accuracy in the test set. These findings underscore the utility of habitat radiomics analysis in characterizing intratumoral heterogeneity and improving early prediction of ALNM.

Accurate preoperative prediction of axillary lymph node metastasis (ALNM) remains a challenge in patients with triple-negative breast cancer (TNBC), given the intratumoral heterogeneity characterized by variable cellularity, necrosis, stromal composition, and perfusion patterns, which are closely linked to invasion and metastatic dissemination (32, 33). This pattern suggests that habitat radiomics features capture complementary ITH information rather than simply providing redundant descriptors of global tumor appearance. From a clinical perspective, axillary lymph node status, washout-type TIC pattern, and high Nottingham grade (G3) were independently associated with ALNM, reflecting underlying aggressive tumor biology. These features are closely linked to key processes such as intratumoral heterogeneity, hypoxia-driven angiogenesis, increased vascular permeability, and high proliferative activity, all of which facilitate invasion and metastatic dissemination. However, the modest performance of the clinical model alone highlights that macroscopic indicators are insufficient to fully capture these complex biological mechanisms, underscoring the need for quantitative imaging biomarkers (34–39).

Recent studies have demonstrated the utility of intratumoral radiomics models derived from MRI in predicting ALNM in breast cancer (40–43). Nevertheless, their predictive capabilities still failed to attain the ideal state. Furthermore, the integration of peritumoral and intratumoral radiomics has been increasingly investigated. Wu et al. (44) developed a fusion nomogram incorporating clinicopathological features and Peri4mm radiomics signatures, achieving high AUC values in the test sets. However, the inclusion of mixed molecular subtypes may obscure TNBC-specific biological characteristics. Moreover, peritumoral regions are inherently susceptible to confounding due to background parenchymal enhancement. Habitat radiomics addresses this limitation by partitioning tumors into subregions with coherent voxel-wise imaging phenotypes, thereby preserving spatially organized microenvironmental niches (16, 45). As highlighted by Maley et al. (46), intratumor heterogeneity is pivotal as it reflects the underlying evolutionary dynamics and microenvironmental niches associated with axillary lymph node metastasis. Building on this, Wu et al. (44) provided evidence in a breast cancer study that habitat-based radiomics, which captures subregional phenotypes, significantly outperforms whole-tumor averaging and peritumoral approaches in predicting axillary lymph node metastasis (ALNM). Chen et al. (28) employed DCE-MRI-based habitat radiomics to predict axillary lymph node metastasis in stage I-II breast cancer. The model achieved AUCs of 0.895, 0.932, and 0.861 in the training, internal validation, and external validation sets, respectively. The superior performance of these large-sample (*n* = 468) habitat research models, which encompass all subtypes of breast cancer, underscores the significance of the habitat model. The application of this habitat model for axillary lymph node metastasis prediction in a single breast cancer subtype remains unexplored.

The diagnostic performance of PET/CT for identifying ALNM has been reported to reach an overall accuracy of 74.1% (47). TNBC is associated with a higher prevalence of PD-L1 expression, which contributes to immune evasion and serves as a predictive biomarker for response to immune checkpoint inhibitors (48). However, the high cost of PET/CT and immunological testing limits their widespread clinical use. Radiomics based on ultrasound and mammography has emerged as a promising method for predicting ALNM due to its non-invasive nature and cost-effectiveness (49, 50). Hong et al. (51) showed that ultrasound-based radiomics exhibits good predictive performance. However, these methods are mainly based on two-dimensional (2D) images and cannot fully capture three-dimensional (3D) spatial information. Deep learning can automatically extract complex and relevant features from raw images (52). Guo et al. (53) reported that a deep learning-based multilayer perceptron (MLP) model achieved an AUC of 0.712 for predicting ALNM. However, deep learning models often lack interpretability due to their end-to-end nature. Overall, existing methods are not comprehensive and largely ignore tumor heterogeneity specific to TNBC. Multiparametric MRI provides a more complete evaluation of tumor heterogeneity by capturing different aspects of tumor biology. T2WI reflects water content and tissue structure. DWI reflects cellular density and diffusion restriction. DCE-MRI reflects perfusion and vascular permeability (54). By focusing on TNBC and incorporating habitat radiomics with improved interpretability, our study addresses this important gap.

In previous studies, the lack of interpretability in machine learning models due to their black-box nature has been a major challenge (55). XGBoost has strong capability in handling high-dimensional and non-linear imaging data and offers relatively good interpretability among machine learning models, as feature importance and individual prediction contributions can be quantified using tree-based explanation methods such as SHAP (56). Previous studies have shown that different machine learning algorithms, including SVM, logistic regression, and XGBoost, demonstrate variable performance across diagnostic tasks, with XGBoost often achieving superior predictive accuracy in radiomics applications (57, 58). SHAP analysis showed that axillary lymph node length was the most important predictor in the model. This finding is in line with clinical practice, as nodal enlargement is a well-known imaging sign of metastatic involvement (59). Previous breast cancer habitat imaging studies have shown that intratumoral habitats may reflect distinct microenvironmental states (60). In this context, habitat subregion 1 was interpreted as the hypoxic–necrotic core, subregion 2 as the transitional zone, and subregion 3 as the highly vascularized and perfused region. The complexity of hypervascular cellular habitat was found to be larger in TNBC compared to non-TNBC, suggesting a higher invasiveness in TNBC (61). Although the habitat subregion 3 feature had a lower SHAP value than lymph node length and the top whole-tumor radiomics feature, this does not mean that habitat-based analysis has limited value. SHAP mainly reflects the individual contribution of each feature in the trained model and tends to assign higher importance to features that are more stable, reproducible, and directly related to the outcome. By contrast, habitat radiomics features are derived from intratumoral subregions and may show greater variability or partial overlap with global radiomics features, which can reduce their individual SHAP values. However, the habitat radiomics model still outperformed the conventional radiomics model, indicating that habitat features provide additional and biologically meaningful information for predicting metastatic risk.

Several limitations should be noted. First, triple-negative breast cancer is relatively rare, so our sample size was limited. This may reduce the stability and generalizability of the results. Second, this was a retrospective study, which may introduce selection bias. In addition, we also did not perform prospective validation, so the clinical value of the model still needs further confirmation. Third, we did not carry out immune-related experiments. Therefore, we could not directly link the imaging features to the tumor immune microenvironment. Future prospective studies with larger cohorts and immune assays are needed.

In conclusion, this study developed an mpMRI-based habitat radiomics model for preoperative prediction of ALNM in TNBC. The habitat radiomics model showed better performance than the clinical and conventional radiomics models, and the combined model achieved the best overall result. These findings suggest that habitat radiomics may provide useful information for ALNM risk assessment, but further prospective validation is still needed.

## Data Availability

The original contributions presented in the study are included in the article/Supplementary material, further inquiries can be directed to the corresponding author/s.
