# Supplementary material for: Multi-parametric MRI habitat radiomics with interpretable machine learning for early prediction of axillary lymph node metastasis in triple-negative breast cancer
Source: Front Med (Lausanne). 2026 May 18;13:1808030. doi: 10.3389/fmed.2026.1808030 (PMC13234856; doi:10.3389/fmed.2026.1808030)
Supplement: Supplementary file 1 [file Data_Sheet_1.docx]

**CONTENTS**

[Supplemental Material S1. Scanning parameters 1](#_Toc227267237)

[Supplemental Material S2. Imaging preprocessing 4](#_Toc227267238)

[Supplemental Material S3. Habitat generation process 9](#_Toc227267239)

[Supplemental Material S4. Feature extraction 20](#_Toc227267240)

[Supplemental Material S5. Feature selection 23](#_Toc227267241)

[Supplemental Material S6. Finding the optimal hyperparameter： 33](#_Toc227267244)

[Supplemental Material S7. Final model training 36](#_Toc227267247)

[Supplemental Material S8 Model Evaluation 43](#_Toc227267253)

[Supplemental Material S9. Model Interpretability Analysis 52](#_Toc227267262)

**Supplemental Material S1. Scanning parameters**

Table 1-1: Detailed MRI parameters

| Parameters | T1WI | T2WI | DWI | DCE |
| --- | --- | --- | --- | --- |
| Sequence | TSE | TSE | SE-EPI | 3D FFE |
| Fat suppression | None | SPAIR | SPAIR | SPAIR |
| Repetition time(ms) | 400 | 5000 | 2500 | 4.5 |
| Echo time(ms) | 10 | 60 | 42 | 2.2 |
| Flip angle (°) | 90 | 90 | 90 | 10 |
| Matrix | 124×131 | 236×279 | 176×167 | 228×301 |
| Field of view(mm) | 248×336 | 248×332 | 354×338 | 272×333 |
| Slice thickness(mm) | 4 | 4 | 4 | 4 |
| Slice gap(mm) | 1.5 | 1.5 | 1.5 | 0 |
| b-values(sec/mm^2^) | None | None | 0/800 | None |

All Patients underwent preoperative MRI using a 3.0 tesla Philips (Netherlands) Achieva dual-gradient superconducting system. They were positioned prone, with both breasts naturally suspended to align with the center of the breast coil. Patients were instructed to hold their breath and maintain stillness to minimize motion artifacts. In the training and test set, the breast MRI sequence and scanning order is as follows: (1) an axial T1-weighted (T1W) with a Turbo spin echo (TSE) sequence and the parameters are as follows: repetition time (TR), 400 ms; echo time (TE),10 ms; slice thickness, 4.0 mm; slice gap, 1.5 mm; field of view (FOV), 248 mm ×336 mm; and matrix, 124 ×131; Flip angle, 90°; (2) an axial T2-weighted (T2W) fat-suppressed with a Turbo spin echo (TSE) sequence and the parameters are as follows: repetition time (TR), 5000 ms; echo time (TE), 60 ms; slice thickness, 4.0 mm; slice gap, 1.5 mm; field of view (FOV), 248 mm ×332 mm; and matrix, 236 ×279; Flip angle, 90°;(3) diffusion-weighted imaging (DWI) with an Spin Echo (SE) of echo planar imaging (EPI) sequence and the parameters are as follows: TR, 2500 ms; TE, 42 ms; slice thickness, 4.0 mm; slice gap, 1.5 mm; FOV, 354 mm × 338 mm; matrix, 176 × 167; and diffusion-sensitive coefficient (b-value) selected at 800 s/mm^2^; Flip angle, 90°; (4) a dynamic contrast-enhanced with 3D Fast Field Echo (3D-FFE) sequence. This sequence consisted of an axial T1-weighted fat-suppressed sequence with the following parameters: TR, 4.5 ms; TE, 2.2 ms; slice thickness, 4 mm; no gap; FOV, 272 mm × 333 mm; and matrix, 228 × 301 Flip angle, 10°. After the axial scan, 15 ml of contrast agent (gadopentetate dimeglumine, Gd-DTPA, flow rate of 2 ml/s, 0.2 mmol/kg) was injected, followed by 20 ml of saline. 23 seconds after the injection, a continuous scan was performed 6 times, with each scan lasting about 60s, for a total scan time of 6 min 23 s.

**Supplemental Material S2. Imaging preprocessing**


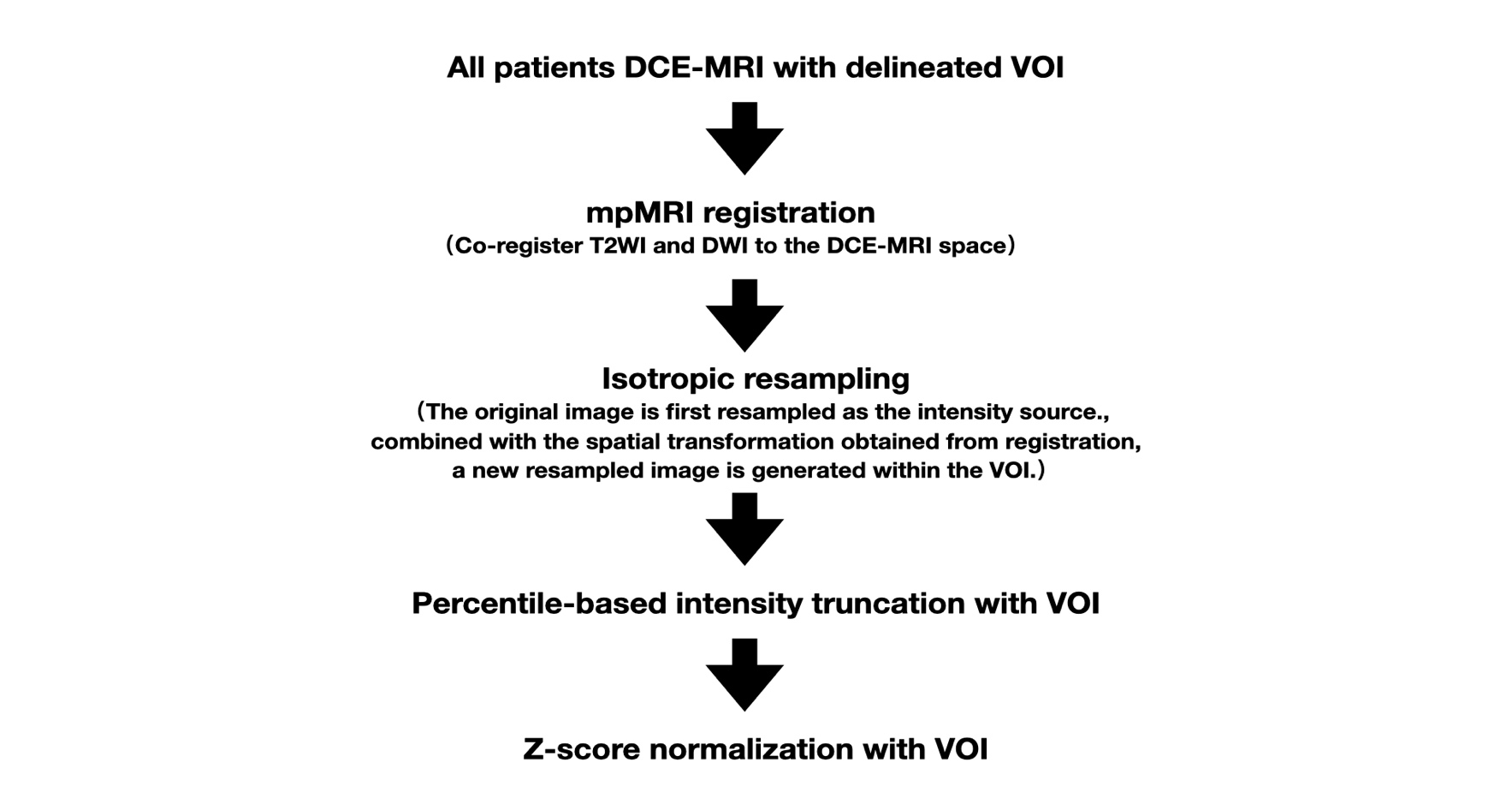


Figure S2-1. Image preprocessing

**1. Multimodal MRI Co-registration**

To ensure robust spatial alignment and voxel-wise correspondence across different MRI modalities prior to habitat extraction, spatial co-registration was strictly modeled as a symmetric diffeomorphic optimization problem. The DCE sequence was designated as the fixed reference space (*I_f*), T2WI, and DWI were treated as moving images (*I_m*).

**1.1 Mathematical Optimization Framework**

The objective of the registration is to find an optimal spatial transformation, denoted as ($\phi^{*}$), that maps the moving image domain to the reference domain. We employed the Symmetric Normalization (*SyN*) algorithm, which optimizes a time-dependent velocity field *v (x, t)* to generate a diffeomorphic mapping. The objective function is formulated as:

$$E\left( \phi\right)=arg{min}_{\phi}[-MI\left( I_{f},I_{m}o\phi\right)+\lambda\int_{0}^{1} \parallel Lv\left( x,t \right)\parallel^{2}dt]$$

where $I_{m}o\phi$ represents the warped moving image, $\lambda$ is the regularization weight, and *L* is a differential operator enforcing smoothness on the velocity field *v*. The mapping $\phi$ is derived by integrating the velocity field over time: $\frac{d\phi}{dt}=v(\phi,t)$, ensuring that the resulting transformation is invertible, smooth, and preserves anatomical topology (*det* ($\nabla\phi)>0$).

**1.2. Similarity Metric for Multimodal MRI**

Given the distinct contrast mechanisms and non-linear intensity relationships between multiparametric MRI sequences, Mutual Information (*MI*) was strictly utilized as the similarity metric rather than Sum of Squared Differences (SSD). *MI* measures the statistical dependency between the reference and moving images, defined by Shannon entropy (*H*):

$$\mathrm{MI}\left( I_{f},I_{m}\circ\phi\right)=H\left( I_{f} \right)+H\left( I_{m}\circ\phi\right)-H(I_{f},I_{m}\circ\phi)$$

This formulation ensures that the optimization process is driven by the shared anatomical boundaries rather than absolute voxel intensities, effectively mitigating cross-modality contrast bias.

**1.3. Algorithmic Implementation (ANTs)**

The mathematical framework was computationally executed using the Advanced Normalization Tools (*ANTsPy*, version 1.8.1) in Python. To avoid local minima, the optimization was performed using a multi-resolution pyramid strategy.

**2. Isotropic Resampling**

To guarantee scale- and rotation-invariance of the extracted radiomic features (particularly texture matrices such as GLCM and GLSZM) in accordance with the Image Biomarker Standardisation Initiative (IBSI) guidelines, all multimodal MRI sequences and their corresponding region-of-interest (ROI) masks were standardized to an isotropic voxel spacing of 1 × 1 × 1 mm³.

**2.1. Mathematical Framework of Grid Transformation**

Medical images are fundamentally discrete representations of continuous physical space. Let $P\in\mathbb{R}^{3}$ denote a physical spatial coordinate vector (x, y, z). The resampling process maps the continuous physical coordinates of the new isotropic grid back to the discrete voxel indices of the original image grid using an affine transformation matrix. The intensity of the new voxel *I_new_(P)* is computed based on the surrounding original voxels *I_old_*.

**2.2 Interpolation Kernels for Images vs. Masks**

Crucially, different interpolation functions were applied to the anatomical images and the binary ROI masks to preserve continuous signal variations and discrete anatomical boundaries, respectively:

**2.2.1 Continuous Image Resampling (Trilinear Interpolation):** For the DCE, T2, and DWI sequences, a 3D linear (trilinear) interpolation kernel was employed to obtain smooth estimates of the new voxel intensities. The new intensity is the weighted sum of the 8 nearest original voxels, where the weight decreases linearly with spatial distance:

$$I_{new}\left( P \right)\sum_{i,j,k\in N\left( P \right)} I_{old}\left( i,j,k \right)\cdot\prod_{d\left\{ x,y,z \right\}} max(0,1-\parallel\triangle d\parallel)=c^{2}$$

This ensures that the physiological contrast gradients of the tumor microenvironment are preserved without introducing artificial high-frequency noise.

**2.2.2 Discrete ROI Mask Resampling (Nearest-Neighbor Interpolation):**

Applying trilinear interpolation to a binary mask (labels 0 and 1) would generate non-integer boundary values (e.g., 0.6), destroying the integrity of the tumor delineation. Therefore, nearest-neighbor interpolation was strictly applied to the ROI masks. The new mask value M_new_(P) takes the label of the closest original voxel:

$$M_{new}(P)=M_{old}(argminV_{i}\parallel P-V_{i}\parallel$$

This mathematically guarantees that the resampled mask remains strictly binary, preventing partial-volume contamination at the tumor-to-parenchyma interface.

**2.3. Algorithmic Implementation**

The spatial resampling pipeline was programmatically executed using the *SimpleITK* library in Python, ensuring sub-millimeter precision in the continuous-to-discrete coordinate transformation. All images were resampled to an isotropic voxel spacing of 1 × 1 × 1 mm³ using trilinear interpolation, while the corresponding ROI masks were resampled using nearest-neighbor interpolation to preserve label integrity. The resampling process was implemented using the *ResampleImageFilter*, with output spacing set to [1, 1, 1], and the original image origin, direction, and size were preserved. Default interpolation parameters were used unless otherwise specified.

**3. Percentile-Based Intensity Truncation with VOI**

Unlike Computed Tomography (CT), Magnetic Resonance Imaging (MRI) intensities lack a standardized physical scale and are highly susceptible to extreme outlier values (e.g., caused by radiofrequency coil artifacts, vascular flow voids, or acquisition noise). Such outliers can severely skew the statistical distribution of the tumor intensities, rendering subsequent parametric standardization (e.g., Z-score) ineffective by compressing the meaningful biological contrast into a narrow dynamic range.

To ensure robust intensity harmonization, a percentile-based truncation (clipping) technique was strictly applied to the voxel intensities within the tumor volume of interest (VOI) prior to normalization.

**3.1 Mathematical Formulation of Outlier Rejection**

Let $\Omega$ denote the set of all voxels spatial coordinates *x* belonging to the tumor VOI, defined by the binary mask *M(x) = 1*. The set of intensity values within the tumor is given by S = I(x)∣x$\in\Omega$

We compute the 1st percentile *S* and the 99th percentile (P_99_) of the empirical cumulative distribution function (eCDF) of *S*. The intensity of every voxel *I(x)* within the image is then transformed into a robustly bounded intensity *I_clipped_(x)* using the following piecewise mathematical clipping function:

$$I_{clipped}(x)=\left\{ \begin{aligned} P_{1}, ifI_{(x)} <P_{1} \\ I_{x},ifP_{1}\leq I_{(x)}\leq P_{99} \\ P_{99},{ifI}_{(x)}>P_{99} \end{aligned} \right.$$

This operation mathematically bounds the signal distribution, effectively neutralising the leverage of spurious extreme voxels while perfectly preserving the linear relationship of the genuine intra-tumoral heterogeneity.

3.2 **Algorithmic Implementation**

This robust statistical truncation was computationally executed using the *NumPy* library in Python. By restricting the percentile calculation exclusively to the tumor VOI (using boolean mask indexing), the thresholds are driven purely by the tumor's physiological properties, avoiding contamination from background air or surrounding healthy fat tissue.

**4. Z-score Normalization with VOI**

Following spatial co-registration, isotropic resampling, and percentile-based truncation, the fundamental problem of arbitrary magnetic resonance imaging (MRI) intensity scales remained. Unlike computed tomography (CT), where radiodensity is anchored to a standardized Hounsfield Unit (HU) scale, raw MRI intensities represent relative tissue magnetization and vary substantially across different scanners, acquisition protocols, and individual patients. To enable biologically meaningful cross-patient and cross-sequence comparisons of radiomic features, a stringent, per-patient and per-sequence intensity normalization was performed using Z-score standardization.

**4.1. Mathematical Formulation of VOI-Specific Standardization**

To prevent the standardized intensities from being biased by irrelevant background noise (e.g., surrounding air) or variable amounts of healthy peripheral tissue, the statistical parameters required for normalization were strictly derived from the intra-tumoral volume of interest (VOI).

Let $\Omega$ represent the set of voxel coordinates *x* belonging exclusively to the tumor VOI, defined by the binary mask M(x) = 1. The intra-tumoral mean intensity ($\mu_{\Omega}$) and standard deviation ($\sigma_{\Omega}$) are defined as:

$$\mu_{\Omega}=\frac{1}{\mid\Omega\mid}\sum_{x\in\Omega} I_{(x)}$$

$$\sigma_{\Omega}=\sqrt{\frac{1}{\mid\Omega\mid}\sum_{x\in\Omega} \left\{ I_{(x)}-\mu_{\Omega} \right\}^{2}}$$

where |$\Omega$| denotes the total number of voxels within the tumor VOI, and $I_{(x)}$ represents the clipped voxel intensity.

The Z-score normalization maps the intensity of each intra-tumoral voxel to a standard normal distribution (centered at zero with unit variance). The normalized intensity *I_norm_(x)* is computed as:

$$I_{norm}(x)=\frac{I\left( x \right)-\mu_{\Omega}}{\sigma\left( \Omega\right)+\epsilon}$$

where $\epsilon$ = 10^-8^ is a strictly positive, infinitesimally small constant added to the denominator to mathematically prevent undefined division-by-zero errors in regions of absolute homogeneity.

**4.2. Algorithmic Implementation**

This VOI-specific standardization was programmatically executed using the *NumPy* library in Python. The operation ensures that the final extracted radiomic features (such as first-order statistics and higher-order texture matrices) accurately reflect the true intra-tumoral biological heterogeneity rather than technical acquisition variations.

**Supplemental Material S3. Habitat generation process**


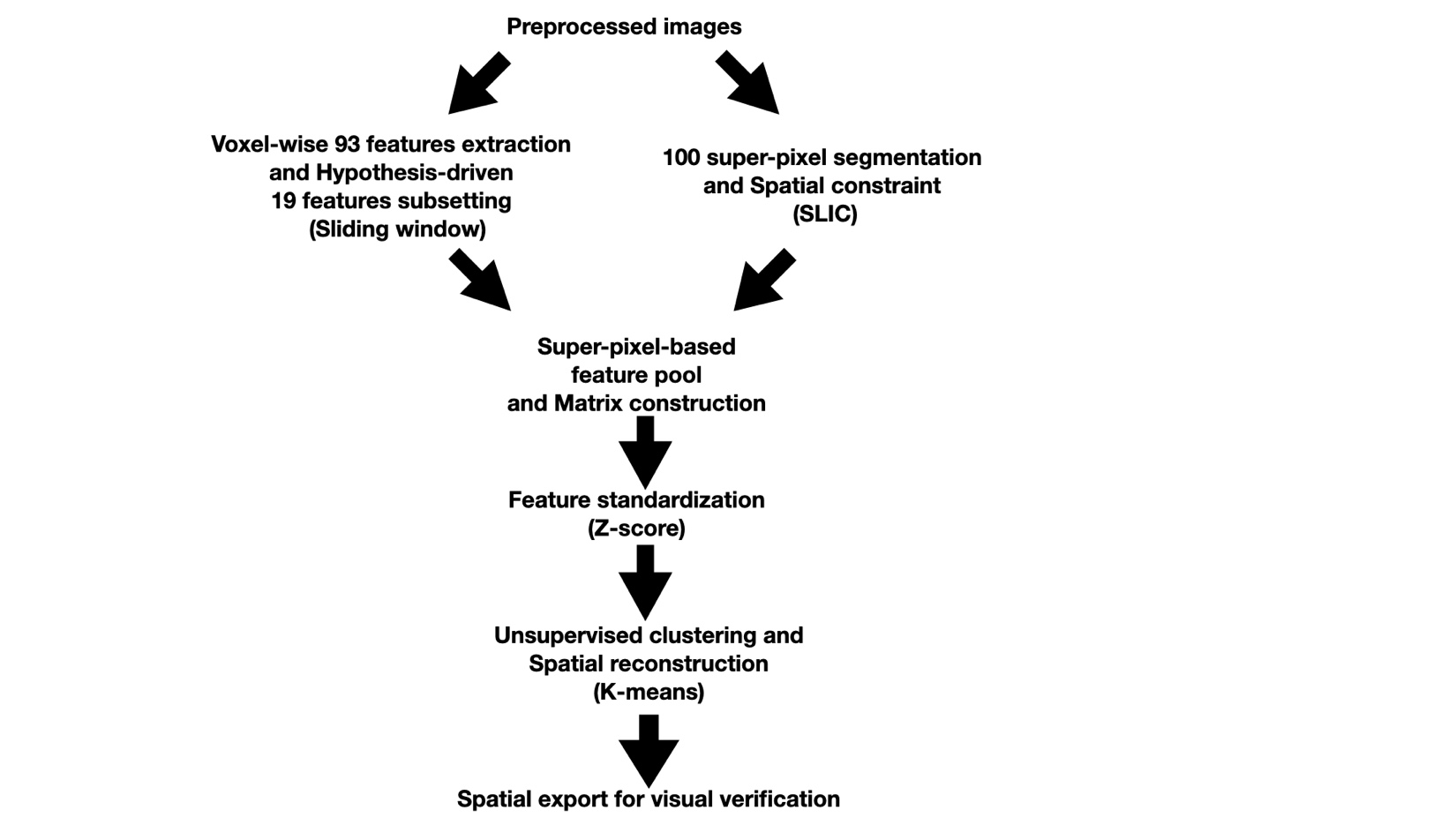


Figure S3-1. Habitat generation pipeline

Our methodology for delineating intratumoral subregions (habitats) was multi-faceted, hypothesis-driven, and involved several rigorous computational steps to ensure both mathematical stability and biological interpretability. The pipeline was executed as follows:

**1. Voxel-wise Feature Extraction via Sliding Window（Feature map generation）**

Initially, to capture the full spectrum of tumoral microscopic heterogeneity, voxel-wise radiomic feature extraction was performed across the entire tumor volume. The voxel-wise radiomic feature maps were generated using a three-dimensional sliding window (3 × 3 × 3 voxels) within the tumor region of interest (ROI).

Specifically, a cubic kernel of 3 × 3 × 3 voxels was centered at each voxel and systematically moved throughout the entire ROI. For each position, radiomic features were calculated based on the local neighborhood defined by the kernel, thereby producing spatially resolved feature maps that characterize intratumoral heterogeneity. These local features constitute the default feature set of the PyRadiomics package, totaling 93. The window size of 3 × 3 × 3 voxels was selected to balance spatial resolution and feature stability. This kernel represents the smallest three-dimensional neighborhood capable of capturing local texture information while preserving fine-grained spatial variations. Larger window sizes may introduce excessive spatial averaging and obscure subtle heterogeneity patterns, whereas smaller neighborhoods may lead to unstable feature estimation. Therefore, the adopted kernel size ensures robust and spatially sensitive quantification of tumor heterogeneity, which is particularly important for subsequent habitat analysis.

Method: voxel-wise radiomic feature extraction was implemented in Python using the open-source PyRadiomics library (version 3.1.0), which is built upon the Insight Segmentation and Registration Toolkit (SimpleITK) for medical image processing.

First, the radiomics feature extractor was initialized using a predefined parameter file (YAML format) via the RadiomicsFeatureExtractor class in PyRadiomics. This parameter file specifies the feature classes to be computed (e.g., first-order statistics and texture features such as firstorder, GLCM, GLRLM, GLSZM, NGTDM and GLDM), as well as key extraction settings. To enable voxel-wise feature computation, the parameter voxelBased was set to True. In this mode, instead of calculating a single feature value for the entire region of interest (ROI), PyRadiomics computes features locally for each voxel using a moving kernel. The size of this kernel is controlled by the parameter kernelRadius. In our implementation, kernelRadius = 1 was used, corresponding to a 3 × 3 × 3 voxel neighborhood (i.e., (2 × radius + 1) ^ 3). The parameter maskedKernel was set to True, ensuring that only voxels within the tumor ROI contribute to feature calculation, thereby avoiding contamination from surrounding non-tumor tissue. The extraction process was executed using the execute function of the PyRadiomics extractor, which takes the input medical image (MRI) and the corresponding binary tumor mask as arguments. Internally, the image and mask are read and processed using SimpleITK, which handles image I/O, spatial metadata, and voxel-wise operations. The output of the extraction is a dictionary-like object containing both scalar features and voxel-wise feature maps. For voxel-based extraction, each radiomic feature is returned as a three-dimensional SimpleITK image, where each voxel intensity represents the computed feature value at that spatial location. Finally, these voxel-wise feature maps were saved to disk in NIfTI format using SimpleITK’s image writing functionality (WriteImage). These feature maps were subsequently used for downstream habitat analysis. This pipeline ensures reproducible and standardized voxel-wise radiomic feature computation and is consistent with prior studies employing PyRadiomics for spatially resolved tumor heterogeneity analysis.

Result: for each voxel, radiomic features were computed from its local neighborhood, resulting in 93 quantitative features（the 93 features are the default feature set extracted by the PyRadiomics package）, including first-order statistics and texture descriptors. This process transformed the original MRI into a set of spatially resolved feature maps, in which each voxel was represented by a high-dimensional feature vector encoding its local imaging phenotype (Table S3-1).

To intuitively illustrate the spatial heterogeneity of the tumor microenvironment prior to clustering, voxel-wise feature maps (heatmaps) (Figure S3-2). were generated for 6 highly representative local features. These heatmaps mapped the quantitative radiomic values back to the original anatomical space, providing a qualitative visual assessment of the spatial distribution and regional variations of key radiomic properties within the tumor.

TableS3-1: 93 quantitative features

| firstorder_10Percentile | firstorder_90Percentile |
| --- | --- |
| firstorder_Energy | firstorder_Entropy |
| firstorder_InterquartileRange | firstorder_Kurtosis |
| firstorder_Maximum | firstorder_Mean |
| firstorder_MeanAbsoluteDeviation | firstorder_Median |
| firstorder_Minimum | firstorder_Range |
| firstorder_RobustMeanAbsoluteDeviation | firstorder_RootMeanSquared |
| firstorder_Skewness | firstorder_TotalEnergy |
| firstorder_Uniformity | firstorder_Variance |
| glcm_Autocorrelation | glcm_ClusterProminence |
| glcm_ClusterShade | glcm_ClusterTendency |
| glcm_Contrast | glcm_Correlation |
| glcm_DifferenceAverage | glcm_DifferenceEntropy |
| glcm_DifferenceVariance | glcm_Id |
| glcm_Idm | glcm_Idmn |
| glcm_Idn | glcm_Imc1 |
| glcm_Imc2 | glcm_InverseVariance |
| glcm_JointAverage | glcm_JointEnergy |
| glcm_JointEntropy | glcm_MaximumProbability |
| glcm_MC | glcm_SumAverage |
| glcm_SumEntropy | glcm_SumSquares |
| gldm_DependenceEntropy | gldm_DependenceNonUniformity |
| gldm_DependenceNonUniformityNormalized | gldm_GrayLevelNonUniformity |
| gldm_DependenceVariance | gldm_GrayLevelVariance |
| gldm_HighGrayLevelEmphasis | gldm_LargeDependenceEmphasis |
| gldm_LargeDependenceHighGrayLevelEmphasis | gldm_LargeDependenceLowGrayLevelEmphasis |
| gldm_LowGrayLevelEmphasis | gldm_SmallDependenceEmphasis |
| gldm_SmallDependenceHighGrayLevelEmphasi | gldm_SmallDependenceLowGrayLevelEmphasis |
| glrlm_GrayLevelNonUniformity | glrlm_GrayLevelNonUniformityNormalized |
| glrlm_GrayLevelVariance | glrlm_HighGrayLevelRunEmphasis |
| glrlm_LongRunEmphasis | glrlm_LongRunHighGrayLevelEmphasis |
| glrlm_LongRunLowGrayLevelEmphasis | glrlm_LowGrayLevelRunEmphasis |
| glrlm_RunEntropy | glrlm_RunLengthNonUniformity |
| glrlm_RunLengthNonUniformityNormalized | glrlm_RunPercentage |
| glrlm_RunVariance | glrlm_ShortRunEmphasis |
| glrlm_ShortRunHighGrayLevelEmphasis | glrlm_ShortRunLowGrayLevelEmphasis |
| glszm_GrayLevelNonUniformity | glszm_GrayLevelNonUniformityNormalized |
| glszm_GrayLevelVariance | glszm_HighGrayLevelZoneEmphasis |
| glszm_LargeAreaEmphasis | glszm_LargeAreaHighGrayLevelEmphasis |
| glszm_LargeAreaLowGrayLevelEmphasis | glszm_LowGrayLevelZoneEmphasis |
| glszm_SizeZoneNonUniformity | glszm_SizeZoneNonUniformityNormalized |
| glszm_SmallAreaEmphasis | glszm_SmallAreaHighGrayLevelEmphasis |
| glszm_SmallAreaLowGrayLevelEmphasis | glszm_ZoneEntropy |
| glszm_ZonePercentage | glszm_ZoneVariance |
| ngtdm_Busyness | ngtdm_Coarseness |
| ngtdm_Complexity | ngtdm_Contrast |
| ngtdm_Strength |  |


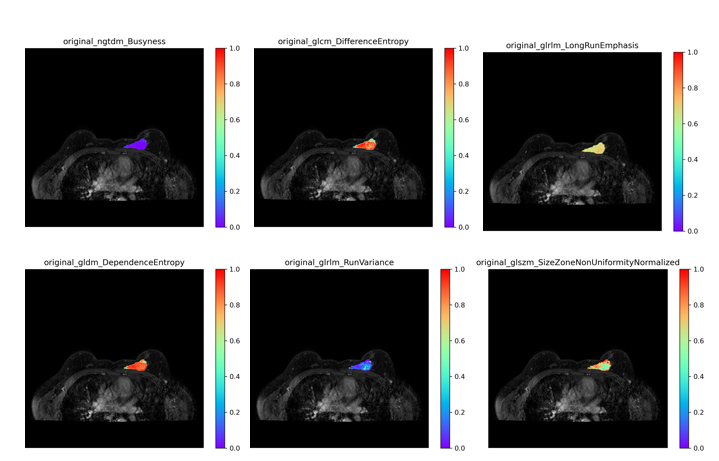


Figure S3-2. quantitative feature heatmap（Take 6 kinds of 93 features as an example）

**2. Hypothesis-Driven Subsetting**

For each voxel within the segmented tumor volume, 93 radiomic features were extracted to characterize local intensity and texture heterogeneity. Due to the typically high correlation and redundancy of high-dimensional voxel-level radiomic features, and to avoid the "curse of dimensionality" as well as prevent overfitting in subsequent unsupervised clustering, we circumvented a purely data-driven approach. Based on prior literature and clinical consensus, a predefined, robust panel of 19 radiomic features was selected for downstream analysis (Table S3-2) ^[1,2,3]^.

Table S3-2. Nineteen radiomics features extracted from each super-pixel.

| Feature subtype | Specific feature name |
| --- | --- |
| Histogram/first-order feature | original_firstorder_Entropy  original_firstorder_MeanAbsoluteDeviation  original_firstorder_Median |
| Texture/high-order feature | original_glcm_DifferenceAverage  original_glcm_DifferenceEntropy  original_glcm_DifferenceVariance  original_glcm_Imc1  original_glcm_Imc2  original_glcm_InverseVariance  original_glcm_JointEnergy  original_glcm_JointEntropy  original_glcm_SumEntropy  original_glrlm_LongRunEmphasis  original_glrlm_RunEntropy  original_glrlm_RunVariance  original_glszm_SizeZoneNonUniformityNormalized  original_glszm_SmallAreaHighGrayLevelEmphasis  original_ngtdm_Contrast  original_ngtdm_Strength |

**3. Super-pixel Segmentation and Spatial Constraint**

We also need to perform super-pixel processing on the original images, which is independent of the voxel-level feature extraction in steps 1 and 2.

Parallel to and independent of the voxel-wise radiomic feature extraction, a spatial constraint track was established directly on the original anatomical images. The objective of this parallel track was to combine intensity similarities with spatial proximity, effectively mitigating inherent voxel-level noise and ensuring the spatial contiguity of the final habitats.

This super-voxel segmentation was executed using Python, utilizing the *segmentation* module within the highly validated *scikit-image* library. We employed the 3D implementation of the Simple Linear Iterative Clustering (SLIC) algorithm. To ensure the segmentation was strictly confined to the tumor parenchyma, the algorithm was executed with the corresponding tumor Region of Interest (ROI) applied as a rigid constraint mask (*mask* parameter).

Algorithmically, the SLIC parameters were rigorously configured to fit the morphological characteristics of the tumor. We parameterized the algorithm to partition the tumor ROI into approximately 100 super-voxels (*n_segments* *= 100*). Crucially, the segmentation was fine-tuned with a compactness parameter set to 10.0 (*compactness = 10.0*). This specific parameter optimally balances the trade-off between the adherence to internal biological intensity boundaries (color/intensity similarity) and the mathematical regularity of the super-voxel shapes (spatial proximity). The output of this independent process was a 3D label mask containing spatially contiguous super-voxels, which subsequently served as the precise spatial boundaries for the pooling of the independently extracted radiomic feature maps.
 The SLIC algorithm clusters pixels based on a combined distance measure of color/intensity and spatial proximity. The core distance function is defined as:

$$D=\sqrt{d_{lab}^{2}+\left( \frac{d_{s}}{S} \right)^{2}}$$

D is the combined distance measure.

d_lab_ is the Euclidean distance in color space (Lab color space).

d_s_ are the Euclidean distance in the image plane.

S is the grid interval or the size of the super-pixel.

**4. Super-pixel-based Feature Pooling and Matrix Construction**

To bridge the highly dimensional voxel-wise feature maps and the subsequent clustering algorithm, a spatial regional aggregation step (mean pooling) was executed. This process was implemented using Python, primarily leveraging the robust multidimensional image processing capabilities of the *NumPy* and *SciPy* libraries.

Specifically, the super-pixel mask generated by the SLIC algorithm consists of discrete integer labels, where each tumor voxel is assigned to a specific spatially contiguous region. For each of the 19 extracted radiomic feature maps, a total of 57 features derived from the three sequences, we spatially overlaid the SLIC mask to extract the corresponding voxel values encompassed within each super-pixel label. The representative feature value for each super-pixel was then computed by calculating the arithmetic mean of these intra-regional voxels using the *scipy. ndimage. mean*. function.

For habitat clustering, each patient was partitioned into **100 supervoxels** within the tumor region. For each supervoxel, **19 imaging features** were extracted from each of the three MRI sequences **(T2WI, DWI, and DCE)**, resulting in a **57-dimensional feature vector** per supervoxel (19×3=57). In the training cohort, **87 patients** were included. Since each patient contributed **100 supervoxels**, the total number of supervoxels used for clustering was: 87×100=8700 Therefore, the feature matrix used for K-means clustering in the training set had a dimension of **8700 × 57**, where: **8700 rows** represented all supervoxels from the 87 training patients, and **57 columns** represented the concatenated multi-sequence features of each supervoxel. Similarly, in the test cohort, **38 patients** were included, yielding: 38×100=3800. Thus, the test feature matrix had a dimension of **3800 × 57**. For feature normalization, each of the 57 features was standardized independently using **Z-score normalization**, with the mean (μ) and standard deviation (σ) estimated from the **training set only**. The same normalization parameters derived from the training cohort were then applied to the test cohort to avoid data leakage.

This spatial pooling operation effectively compressed and transformed the volumetric 3D feature maps into a highly structured 2D features matrix. In this matrix, each row represents an individual super-pixel habitat candidate, and each column represents a specific radiomic feature. This robust 8700 times 57 matrix served as the definitive, noise-reduced input for the subsequent Z-score standardization and K-means clustering steps.

Let the standardized data matrix be denoted as:

$Z\in\mathbb{R}^{n\times p}$ (*n* = 8700, *p* = 57)

**5. Feature Standardization in training set**

Prior to the K-means clustering analysis, it was mathematically imperative to eliminate dimensional biases. The 57 pooled radiomic features inherently possess vastly different numerical scales and units (e.g., fractional values for Entropy versus thousands for Energy). Because K-means is a distance-based algorithm relying on Euclidean geometry, failing to normalize these inputs would cause features with larger absolute magnitudes to disproportionately dominate the distance calculations, thereby eclipsing the contributions of subtle but biologically critical texture features. To construct an equitable multidimensional feature space, we applied a strict Z-score standardization. This normalization process was computationally executed using the *preprocessing* module within the *scikit-learn* Python library, specifically employing the *StandardScaler* class.

The standardization was applied strictly column-wise across the N ×57 pooled feature matrix (where N represents the number of spatially constrained super-pixels, N =8700). For each of the 57 distinct radiomic features, the algorithm computed the sample mean ($\sigma_{j}$) and the standard deviation ($\mu_{j}$) across all super-pixels. Each raw pooled feature value ($x_{ij}$) was then mathematically transformed into its standardized form ($Z_{ij}$) utilizing the following equation:

Z-score normalization:

$$Z_{ij}=\frac{x_{ij}-\mu_{j}}{\sigma_{j}}$$

where:

$x_{ij}$ is the value of the *j*-th feature for voxel *i*,

$\mu_{j}$ and $\sigma_{j}$ are the mean and standard deviation of feature *j*, respectively.

This transformation ensures that each feature has zero mean and unit variance, preventing features with large numerical ranges from dominating the analysis.

Algorithmically, the scaler was configured with its core parameters explicitly set to center the data (*with_mean = True*) and scale the data to unit variance (*with_std = True*). Consequently, all 57 feature columns were perfectly harmonized to possess a mean of 0 and a standard deviation of 1. This rigorous normalization effectively neutralized the dimensional discrepancies, providing a robust, scale-invariant input matrix for the subsequent unsupervised habitat clustering.

**5. Unsupervised Clustering and Spatial Reconstruction of Intratumoral Habitats**

Following the rigorous spatial pooling and Z-score standardization, the 8700×57-dimensional feature matrix was subjected to unsupervised clustering to identify distinct, cohesive intratumoral habitats. This was computationally executed using the K-means algorithm implemented via the *cluster* module of the *scikit-learn* Python library.

**Algorithm Configuration and Optimization:** To ensure robust and reproducible clustering, the algorithm was configured with the *init='k-means++'* parameter, which intelligently accelerates convergence and avoids suboptimal local minima by spatially distributing the initial cluster centroids. To further guarantee stability, the algorithm was executed with 10 independent random initializations (*n_init=10*), and a fixed *random_state* was applied to ensure the exact reproducibility of the subregions. The K-means algorithm is a method for clustering data into K distinct clusters. The algorithm iteratively updates the centroids of each cluster to minimize the within-cluster sum of squares. The key formula for K-means is the objective function to be minimized:

$$J=\sum_{i=1}^{N} \sum_{k=1}^{K} w_{ik}\times\parallel x_{i}-\mu_{k}\parallel^{2}$$

*J* is the objective function.

*N* is the number of data points.

*K* is the number of clusters.

*w_ik_* is a binary indicator (1 if data point *i* is in cluster *k*, 0 otherwise).

*x_i_* is the Habitat data point.

*μ_k_* is the centroid of cluster *k*.

∥ *x_i_-μ_k_* ∥^2^ is the squared Euclidean distance between data point *i* and centroid *k*.

To mathematically objectively determine the optimal number of habitats (*K*) and prevent arbitrary assignment, we iteratively evaluated *K* values ranging from 2 to 10. For each spatial configuration, the clustering performance was strictly quantified using the **Silhouette Coefficient** via the *metrics. silhouette_score* function in *scikit-learn*.

The Silhouette Coefficient is a highly interpretable index ranging from -1 to 1 that simultaneously evaluates intra-cluster cohesion and inter-cluster separation. For each individualized super-pixel *i*, its silhouette value *s(i)* is mathematically defined as:

$$S(i)=\frac{b\left( i \right)-a(i)}{max(a\left( i \right),b\left( i \right))}$$

where a(*i*) represents the mean Euclidean distance between super-pixel *i* and all other super-pixels assigned to the same habitat (intra-cluster mean distance), effectively measuring cluster compactness. Conversely, b(*i*) represents the minimum mean distance between super-pixel *i* and all super-pixels in the nearest neighboring habitat (inter-cluster mean distance), measuring cluster separation.

The global Silhouette score for a specific *K* configuration is computed as the arithmetic mean of *S(i)* across all $N$ super-pixels. A higher global Silhouette score indicates that the defined habitats are dense, well-separated, and distinct in their radiomic phenotypes. Consequently, the specific *K* value =3 that yielded the maximum global Silhouette score was selected as the optimal biological configuration, dictating the final number of subregions for the 3D habitat reconstruction (Figure S3-3).

**Spatial Reconstruction of 3D Habitats:** Crucially, after K-means assigned a discrete habitat label (e.g., Habitat 1, 2, or 3) to each of the N rows in the feature matrix, these mathematical vectors had to be projected back into the original anatomical space. A reverse-mapping operation was performed using multidimensional array broadcasting in *NumPy*. We iterated through the original 3D SLIC super-voxel mask; for each unique super-voxel identifier, its corresponding constituent voxels in the 3D space were uniformly reassigned the newly computed K-means habitat label. This process effectively transformed the 1D cluster array back into a fully registered, 3D anatomical NIfTI mask, wherein distinct tumor subregions (habitats). Specifically, for each patient, all supervoxels assigned to the same cluster were merged to generate a patient-specific habitat mask corresponding to that subregion. In this way, three habitat subregions were obtained for each patient on the original image. These habitat masks were then used as subregional ROIs for subsequent feature extraction and quantitative analysis.


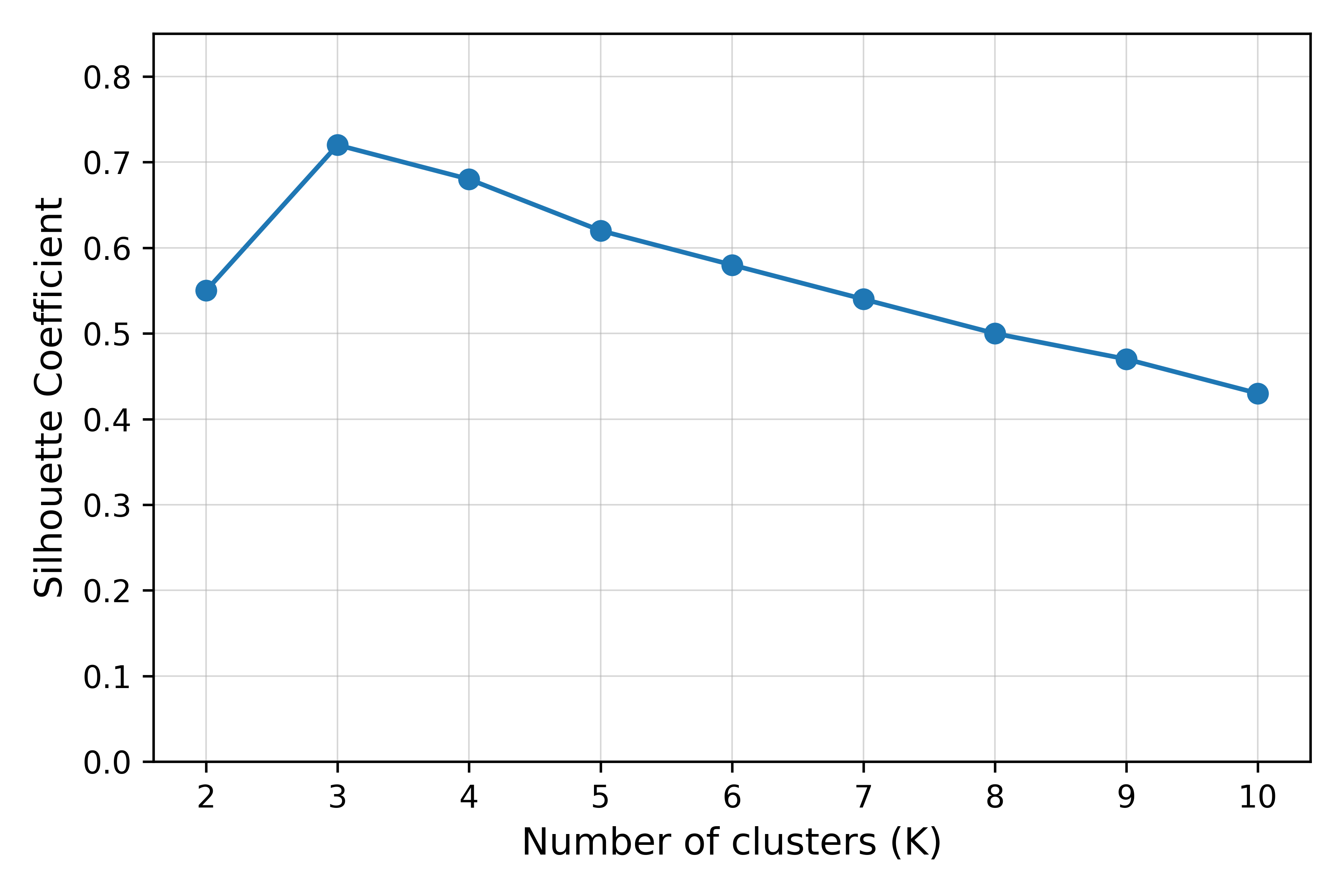


**Supplemental Material Figure S3-3:** Silhouette analysis for determining the optimal number of clusters. The average silhouette coefficient was calculated for different cluster numbers (K). The maximum average silhouette value was obtained at K = 3, indicating the best balance between intra-cluster compactness and inter-cluster separation; therefore, K = 3 was selected for subsequent clustering analysis.

**7. Spatial Export and NIfTI Formatting for Visual Verification**

To translate the mathematically computed 3D habitat matrix back into a clinically interpretable format, the raw 3D NumPy array was exported as a standard NIfTI file (*. nii.gz*). This spatial export was critically handled using the *SimpleITK* Python library to ensure absolute spatial fidelity and anatomic alignment.

In medical imaging, volumetric matrices must maintain strict physical coordinate systems to perfectly register with the native anatomical images. Therefore, the discrete habitat matrix was first converted into a SimpleITK Image object. Subsequently, a rigorous metadata duplication process was executed: the physical ***Origin***, voxel ***Spacing***, and ***Direction Cosine Matrix*** were directly extracted from the patient's native sequence and explicitly copied to the newly generated habitat image using the *CopyInformation* operation. This meticulous spatial alignment ensured that the exported 3D subregions accurately overlay the original tumor parenchyma without any spatial shifts or rotational distortions. The final aligned.*nii.gz* files were thereby primed for downstream intuitive visualization, 3D rendering, and qualitative clinical assessment via dedicated medical image viewers (e.g., *ITK-SNAP*).

Refference

[1] Zhang, B., Zhang, S., Tian, G., Wang, H., Zhu, D., Ban, Y., & Yang, B. (2026). MRI-based Intratumoral and Peritumoral Habitat Radiomics for Early Prediction of Pathologic Complete Response in Breast Cancer: A Multicenter Study. Academic radiology, 33(3), 889–899. https://doi.org/10.1016/j.acra.2025.12.012

[2] Chen, H., Liu, Y., Zhao, J., Jia, X., Chai, F., Peng, Y., Hong, N., Wang, S., & Wang, Y. (2024). Quantification of intratumoral heterogeneity using habitat-based MRI radiomics to identify HER2-positive, -low and -zero breast cancers: a multicenter study. Breast cancer research: BCR, 26(1), 160. https://doi.org/10.1186/s13058-024-01921-7

[3] Zhu, J., Xu, B., Fan, T., Ji, S., Gu, K., Ding, J., Lu, H., Ma, J., & Zhou, Y. (2025). Sub-regional radiomics combining multichannel 2-dimensional or 3-dimensional deep learning for predicting neoadjuvant chemo-immunotherapy response in esophageal squamous cell carcinoma: a multicenter study. NPJ precision oncology, 9(1), 248. https://doi.org/10.1038/s41698-025-01047-9

**Supplemental Material S4. Feature extraction**

Table S4-1: Exection features

| Image Type | Shap | First-order | GLCM | GLRLM | GLSZM | GLDM | NGTDM | Subtotal |
| --- | --- | --- | --- | --- | --- | --- | --- | --- |
| Original (1) | 14 | 18 | 24 | 16 | 16 | 14 | 5 | 107 |
| Wavelet (8) | - | 18 | 24 | 16 | 16 | 14 | 5 | 744 (93×8) |
| LoG (2) | - | 18 | 24 | 16 | 16 | 14 | 5 | 186(93×2) |
| Total Features | 14 | 198 | 264 | 176 | 176 | 154 | 55 | 1037 |

**(1) Shape features** (n=14) were exclusively extracted from the original image, as their values remain invariant across different filter transformations. Including: **Elongation; Flatness; LeastAxisLength; MajorAxisLength; MinorAxisLength; Sphericity; SurfaceArea; SurfaceVolumeRatio; VoxelVolume; MeshVolume; Maximum2DDiameterColumn; Maximum2DdiameterRow; Maximum2DdiameterSlice; Maximum3Ddiameter**

(2) **First-order** (n=18) Including: Energy; Entropy; Minimum; Maximum; Mean; Median; Range; InterquartileRange; MeanAbsoluteDeviation; RobustMeanAbsoluteDeviation; RootMeanSquared; StandardDeviation; Skewness; Kurtosis; Variance; Uniformity; 10Percentile; 90Percentile

(3) **texture features**

GLCM n=24, (Autocorrelation; JointAverage; ClusterProminence; ClusterShade; ClusterTendency; Contrast; Correlation; DifferenceAverage; DifferenceEntropy; DifferenceVariance; JointEnergy; JointEntropy; Id; Idm; Idmn; Idn; Imc1; Imc2; InverseVariance; MaximumProbability; SumAverage; SumEntropy; SumSquares; SumVariance)

GLRLM n=16, (ShortRunEmphasis; LongRunEmphasis; GrayLevelNonUniformity; GrayLevelNonUniformityNormalized; RunLengthNonUniformity; RunLengthNonUniformityNormalized; RunPercentage; GrayLevelVariance; RunVariance; RunEntropy; LowGrayLevelRunEmphasis; HighGrayLevelRunEmphasis; ShortRunLowGrayLevelEmphasis; ShortRunHighGrayLevelEmphasis; LongRunLowGrayLevelEmphasis; LongRunHighGrayLevelEmphasis)

GLSZM n=16, (SmallAreaEmphasis; LargeAreaEmphasis; GrayLevelNonUniformity; GrayLevelNonUniformityNormalized; SizeZoneNonUniformity; SizeZoneNonUniformityNormalized; ZonePercentage; GrayLevelVariance; ZoneVariance; ZoneEntropy; LowGrayLevelZoneEmphasis; HighGrayLevelZoneEmphasis; SmallAreaLowGrayLevelEmphasis; SmallAreaHighGrayLevelEmphasis; LargeAreaLowGrayLevelEmphasis; LargeAreaHighGrayLevelEmphasis)

GLDM n=14, (SmallDependenceEmphasis; LargeDependenceEmphasis; GrayLevelNonUniformity; DependenceNonUniformity; DependenceNonUniformityNormalized; GrayLevelVariance; DependenceVariance; DependenceEntropy; LowGrayLevelEmphasis; HighGrayLevelEmphasis; SmallDependenceLowGrayLevelEmphasis; SmallDependenceHighGrayLevelEmphasis; LargeDependenceLowGrayLevelEmphasis; LargeDependenceHighGrayLevelEmphasis)

NGTDM n=5; (Coarseness; Contrast; Busyness; Complexity; Strength)

total n=93 per image were extracted from the original image and all 10 transformed images (8 Wavelet decompositions and 2 LoG filters).

**Wavelet decompositions (n=8):** wavelet-LLL; wavelet-LLH; wavelet-LHL; wavelet-LHH; wavelet-HLL; wavelet-HLH; wavelet-HHL; wavelet-HHH

**LoG filters (n=2):** log-sigma-3-0-mm-3D; log-sigma-4-0-mm-3D

Wavelet and LoG filters were used to enhance radiomic characterization of breast cancer habitats at multiple spatial scales. Wavelet decomposition captures both low-frequency structural information and high-frequency textural heterogeneity, whereas LoG filtering emphasizes local intensity transitions and edge-related patterns after noise suppression. Wavelet features often account for a large proportion of selected features because they provide rich multiscale information and markedly expand the candidate feature pool. The LoG sigma parameter was chosen according to the spatial scale of interest, with smaller sigma values emphasizing fine details and larger values highlighting coarser heterogeneity; smaller sigma values (e.g., 1–2 mm) mainly capture fine-scale intensity variations and are more susceptible to noise, especially in MRI, potentially leading to reduced feature robustness, therefore, it was not adopted.

A total of 1037 candidate radiomics features were ultimately generated for subsequent feature selection.

Specific Methods：

Radiomics feature extraction was performed using the *PyRadiomics* library implemented in *Python*. Image preprocessing and I/O operations were conducted using the *SimpleITK* package. All MRI sequences (T2WI, DWI, and DCE) and corresponding segmentation masks were first resampled to an isotropic voxel spacing of 1 × 1 × 1 mm³ using 3D linear (trilinear) interpolation for images and nearest-neighbor interpolation for masks to ensure spatial consistency. A *RadiomicsFeatureExtractor* was initialized with predefined parameters, including resampling settings, gray-level discretization, and enabled feature classes and image types. In addition to original images, Laplacian of Gaussian (LoG) filters with σ values of 3.0 mm and 4.0 mm were applied to generate filtered images (log-sigma-3-0-mm-3D and log-sigma-4-0-mm-3D), allowing multi-scale feature extraction. Feature extraction was conducted in a hierarchical manner. For each patient, all three MRI sequences were processed independently. Within each sequence, features were extracted from four regions of interest (ROIs), including the whole tumor and three predefined habitat subregions. For each combination of patient, sequence, and ROI, the function *extractor. execute (image, mask)* was used to compute features within the corresponding mask. To ensure traceability, feature names were systematically organized by incorporating sequence type, ROI, and image transformation. All extracted features were aggregated into a patient-level feature matrix, where each row corresponded to one patient and each column represented a radiomics feature. This matrix was subsequently used for downstream analysis, including feature selection, model training, and independent validation.

**Supplemental Material S5. Feature selection**

**(Using the integration of all whole-tumor and habitat features selection as an example)**


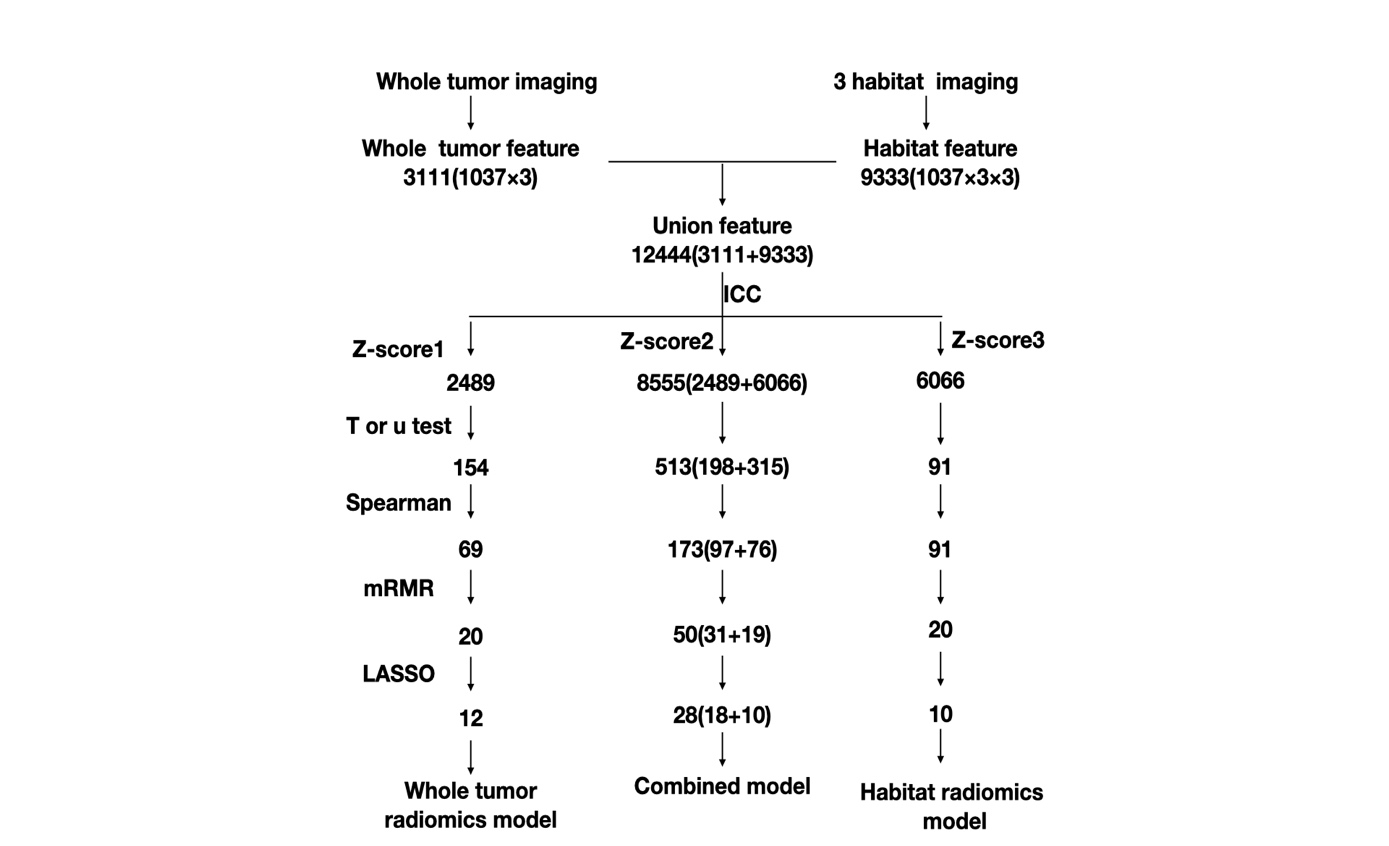


**Figure S5-1: 3 radiomics model feature selation**

**1. ICC in all patients feature**

To assess robustness against segmentation variability, ICC analysis was performed on all extracted radiomic features on a feature-by-feature basis, including both whole-tumor features and habitat-based subregional features. Only features with ICC > 0.90 were retained. A higher ICC indicates that the variability of a feature is mainly due to ***true inter-patient differences***, rather than segmentation inconsistency between readers. Given that the same two raters evaluated all subjects and the aim was to assess absolute agreement between measurements, ICC was calculated using a two-way random-effects model for absolute agreement of single measurements, i.e., ICC (2,1). Under this model, for a feature measured in *n* subjects by *k* raters, the ICC is estimated as:

$$ICC(2,1)=\frac{{MS}_{s}-{MS}_{E}}{{MS}_{s}+\left( k-1 \right)+\left( \frac{k}{n} \right)({MS}_{R}-{MS}_{E})}$$

where *MS_s_*, *MS_R_*, and *MS_E_* denote the mean squares for subjects, raters, and residual error, respectively.

ICC screening was performed independently for each feature, regardless of whether it belonged to the whole-tumor or habitat-based feature set. Specifically, ICC screening was applied to 3111 whole-tumor features (1037 features × 3 MRI sequences) and 9333 habitat-based features (1037 features × 3 MRI sequences × 3 habitats). Features with **ICC > 0.90** were considered highly reproducible and retained for downstream analysis.

For feature *f*, let the feature value extracted from the segmentation of the *i*-th patient by the *j*-th observer be $x_{ij}^{(f)}$, then:

$$\mathcal{X}^{(f)}=\left\{ \mathcal{X}_{ij}^{(f)}\mid i=1\ldots.125;j=1,2 \right\}$$

Calculate each feature *f* separately:

$${ICC}^{(f)}=ICC(\mathcal{X}^{\left( f \right)})$$

The final retained feature set is:

$$\mathcal{F}_{robust}=\left\{ f\in\mathcal{F\mid}{ICC}^{\left( f \right)}>0.90 \right\}$$

Result: Table S5-1 After ICC retained features

Table S5-1: After ICC retained features

| Feature group | Totall | ICC>0.90 Retained |
| --- | --- | --- |
| Whole tumor | 3111 | 2489 |
| Habitat | 9333 | 6066 |
| Overall | 12444 | 8555 |

**2. Alone Z-score normalization with every feature in training set**

Rationale for Z-score normalization: after interobserver reproducibility analysis, 2489 conventional whole-tumor radiomic features and 6066 habitat-based radiomic features remained for further analysis. Because these radiomic features were derived from different MRI sequences, image transformations, and feature families, their numerical ranges varied substantially. For example, first-order intensity features, texture features, and filtered high-order features may differ by several orders of magnitude. Directly comparing or modeling such features without scaling may lead to biased feature selection and unstable model fitting

2.2. Mathematical principle of Z-score normalization

For a given feature $\mathcal{X}_{j}$, let:

$\mathcal{X}_{ij}$ denote the value of feature *j* for patient *i*

$\mu_{j}$denote the mean value of feature *j* in the training set

$\sigma_{j}$denote the standard deviation of feature j in the training set

$$\mu_{j}=\frac{1}{n}\sum_{i=1}^{n} \mathcal{X}_{ij}$$

$$\sigma_{j}=\sqrt{\frac{1}{n-1}\left\{ \sum_{i=1}^{n} \left( \mathcal{X}_{ij}-\mu_{j} \right)^{2} \right\}}$$

Z-score normalization:

$$Z_{ij}=\frac{x_{ij}-\mu_{j}}{\sigma_{j}}$$

where:

$x_{ij}$ is the value of the *j*-th feature for voxel *i*,

$\mu_{j}$ and $\sigma_{j}$ are the mean and standard deviation of feature *j*, respectively.

After transformation, each feature has: mean approximately equal to 0; standard deviation approximately equal to 1. This standardization preserves the relative differences among patients while removing scale-related effects across features.

The parameters for Z-score normalization were estimated using the training set only. For each feature, the mean and standard deviation were calculated from the training cohort and used to standardize the training data. The same parameters were subsequently applied to the test cohort, rather than recalculated within that cohort, to avoid data leakage.

In Python, Z-score normalization can be implemented using the *StandardScaler* function from the *scikit-learn* package.

**3. *T-*test or *Mann-Whitney U test* with each feature in training set**

Univariate feature screening was conducted based on the **training-set radiomics matrix**, in which **rows corresponded to patients** and **columns corresponded to radiomic features**. Rather than analyzing the entire matrix, each feature column was examined separately. For each radiomic feature, patients were divided into two groups according to the binary clinical outcome, and the distribution of that feature was compared between the two groups.

Depending on the distributional characteristics of the feature, either the **independent-samples *Student’s t-test*** or the ***Mann–Whitney U test*** was used. Features showing a significant difference between the two groups **(*P* < 0.05)** were retained for further analysis. Statistical analyses were implemented in ***R*** using the *t.test ()* and *wilcox.test ()* functions from the *stats* **package**.

Result: Table S5-2 After *T* or *U* test retained features

Table S5-2 After *T* or *U* test retained features

| Feature group | Totall | ICC>0.90 Retained | *T* or *U* teat < 0.05 Retained |
| --- | --- | --- | --- |
| Whole tumor | 3111 | 2489 | 198 |
| Habitat | 9333 | 6066 | 315 |
| Overall | 12444 | 8555 | 513 |

**4. Spearman correlation filtering**

To eliminate redundant information and reduce multicollinearity, Spearman correlation analysis was conducted on the training dataset. The Spearman rank correlation coefficient (ρ) was computed for all pairwise combinations of radiomic features using the “*pandas*” library in *Python*. A correlation matrix was generated, and for any pair of features with a high correlation (|ρ| > 0.9), one feature was excluded. Specifically, the feature with the lower correlation with the target variable was removed to retain the most informative features. This procedure ensured that the selected features were relatively independent and minimized redundancy.

Result: Table S5-3 After Spearman retained features

Table S5-3 After Spearman retained features

| Feature group | Totall | ICC>0.90 Retained | *T* or *U* teat < 0.05 Retained | Spearman \|*ρ*\| > 0.9 Retained |
| --- | --- | --- | --- | --- |
| Whole tumor | 3111 | 2489 | 198 | 97 |
| Habitat | 9333 | 6066 | 315 | 76 |
| Overall | 12444 | 8555 | 513 | 173 |

**5. mRMR in training set**

To mitigate the curse of dimensionality and prevent the model from overfitting, a two-step feature selection process was strictly performed **on the training set only**, preventing any potential data leakage to the testing cohort. In the first step, the Minimum Redundancy Maximum Relevance (mRMR) algorithm was employed to eliminate redundant and irrelevant radiomics features. The mRMR algorithm ranks features by maximizing the mutual information between the features and the target variable (maximum relevance, *D*) while simultaneously minimizing the mutual information among the features themselves (minimum redundancy, *R*). The objective function is defined as maximizing the mutual information difference: max (*D* - *R*). Through this filter-based approach, the high-dimensional radiomics feature space was compressed, and the top 50 optimal features with the highest objective scores were retained. This optimized subset of features was subsequently utilized as the input for the Least Absolute Shrinkage and Selection Operator (LASSO) regression model. All mRMR computations were implemented using the *mRMRe* package in *R* software.

Result: Table S5-4 After mRMR retained features

Table S5-4 After mRMR retained features

| Feature group | Totall | ICC>0.90 Retained | *T* or *U* teat < 0.05 Retained | Spearman \|*ρ*\| > 0.8 Retained | mRMR Retained |
| --- | --- | --- | --- | --- | --- |
| Whole tumor | 3111 | 2489 | 198 | 97 | 31 |
| Habitat | 9333 | 6066 | 315 | 76 | 19 |
| Overall | 12444 | 8555 | 513 | 173 | 50 |

**6. LASSO in training set**

To reduce feature redundancy and prevent overfitting, feature selection was performed using a least absolute shrinkage and selection operator (LASSO) model with L1 regularization.

First, all radiomics features were z-score standardized to ensure comparability across different scales. Let $X\in\mathbb{R}^{n\times p}$denote the feature matrix, where *n* is the number of samples and *p* is the number of extracted radiomic features, and let $\mathcal{Y\in}\mathbb{R}^{n}$ denote the corresponding outcome variable.

In training set, to determine the optimal penalization parameter *λ*, a 10-fold cross-validation procedure was implemented: The dataset was randomly partitioned into 10 approximately equal-sized subsets. For each candidate value of *λ*, the model was trained on 9 subsets and validated on the remaining subset. This process was repeated 10 times, with each subset serving as the validation set once. The cross-validation error was computed as the average prediction error across all folds. For regression tasks, the mean squared error (MSE) was used as the evaluation metric:

$$MSE\left( \lambda\right)=\frac{1}{10}\left\{ \sum_{k=1}^{10} \frac{1}{n_{k}}\sum_{i\in\mathcal{D}_{k}} \left\{ y_{i}-y_{i}^{-k}\left( k \right) \right\}^{2} \right\}$$

where$\mathcal{D}_{k}$ denotes the *k*-th validation fold,$n_{k}$ is the number of samples in that fold, and$y_{i}-y_{i}^{-k}\left( k \right)$is the prediction for sample *i* obtained from the model trained without the *k*-th fold.

**One-standard-error rule (*λ*_1se):** the largest λ within one standard error of the minimum error, resulting in a more parsimonious model. The optimal *λ* was selected based on the minimum criterion, and the final LASSO model was refitted using the entire dataset at this *λ*. Features with non-zero coefficients in the final model were retained as the selected radiomic features. These features were subsequently used for downstream model construction and analysis.

Specifically, a 10-fold cross-validation was conducted to determine the optimal penalization parameter ($\lambda$). The cross-validation error curve (**Figure S5-2**) identifies the ($\lambda$) value that minimizes the mean squared error (or deviance).

Second, the LASSO coefficient path (**Figure S5-3**) illustrates the dynamic shrinkage of feature weights under *L_1* regularization. By applying the optimal ($\lambda$) obtained from the first step (indicated by the vertical line), the majority of irrelevant or redundant radiomics features were penalized to zero.

Finallsy, this process successfully yielded exactly 28 non-zero feature coefficients. The specific weights and polarities of these 28 retained features are explicitly presented in the bar chart (**Figure 5-4, Table S5-6**), which constitute the final, highly interpretable radiomics signature formula (Rad_score).


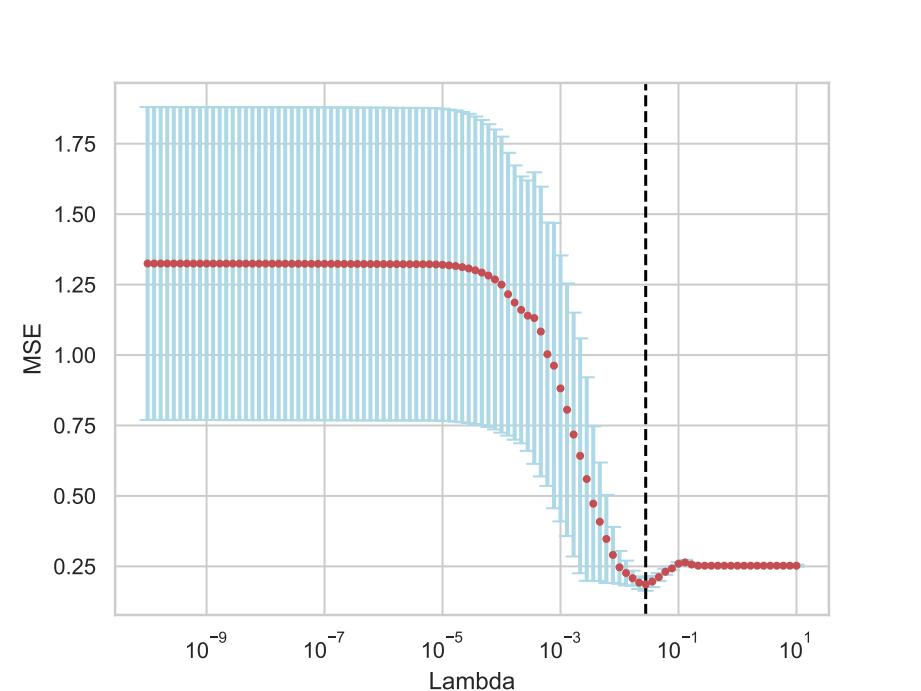


Figue S5-2: LASSO regression 10-fold cross-validation plot, where the red dots represent the mean of the mean squared error (MSE), the blue line represents the confidence interval, and the vertical dashed line indicates the optimal regularization parameter (𝜆) selected by cross-validation, at which point the LASSO regression model achieves the minimum MSE and relatively highest accuracy.


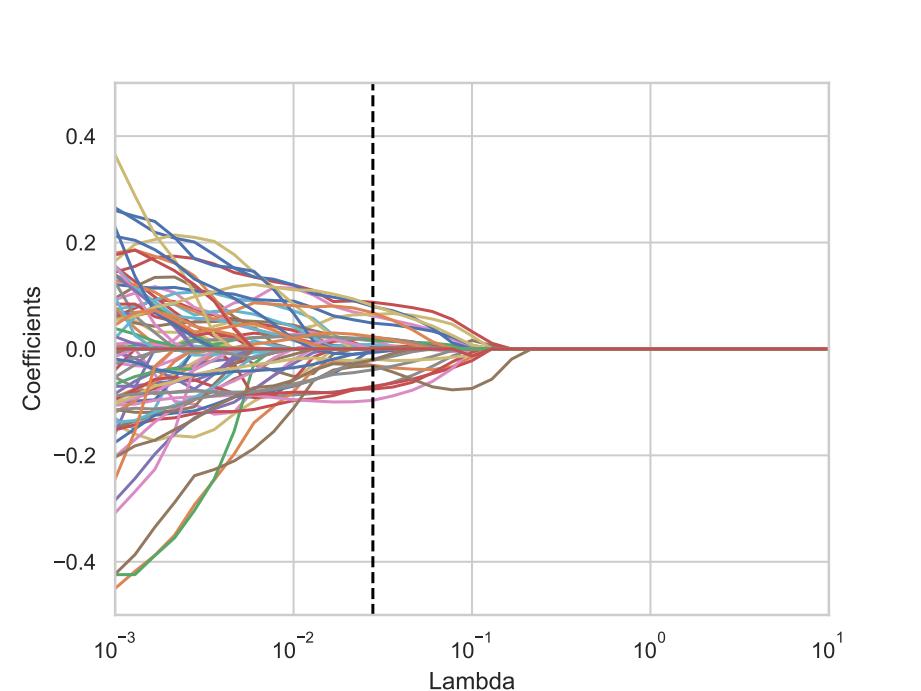


Figue S5-3: LASSO regression feature coefficient plot, as 𝜆 increases, the feature coefficients are continuously compressed until they reach zero. At the optimal 𝜆, 28 feature coefficients remain non-zero.


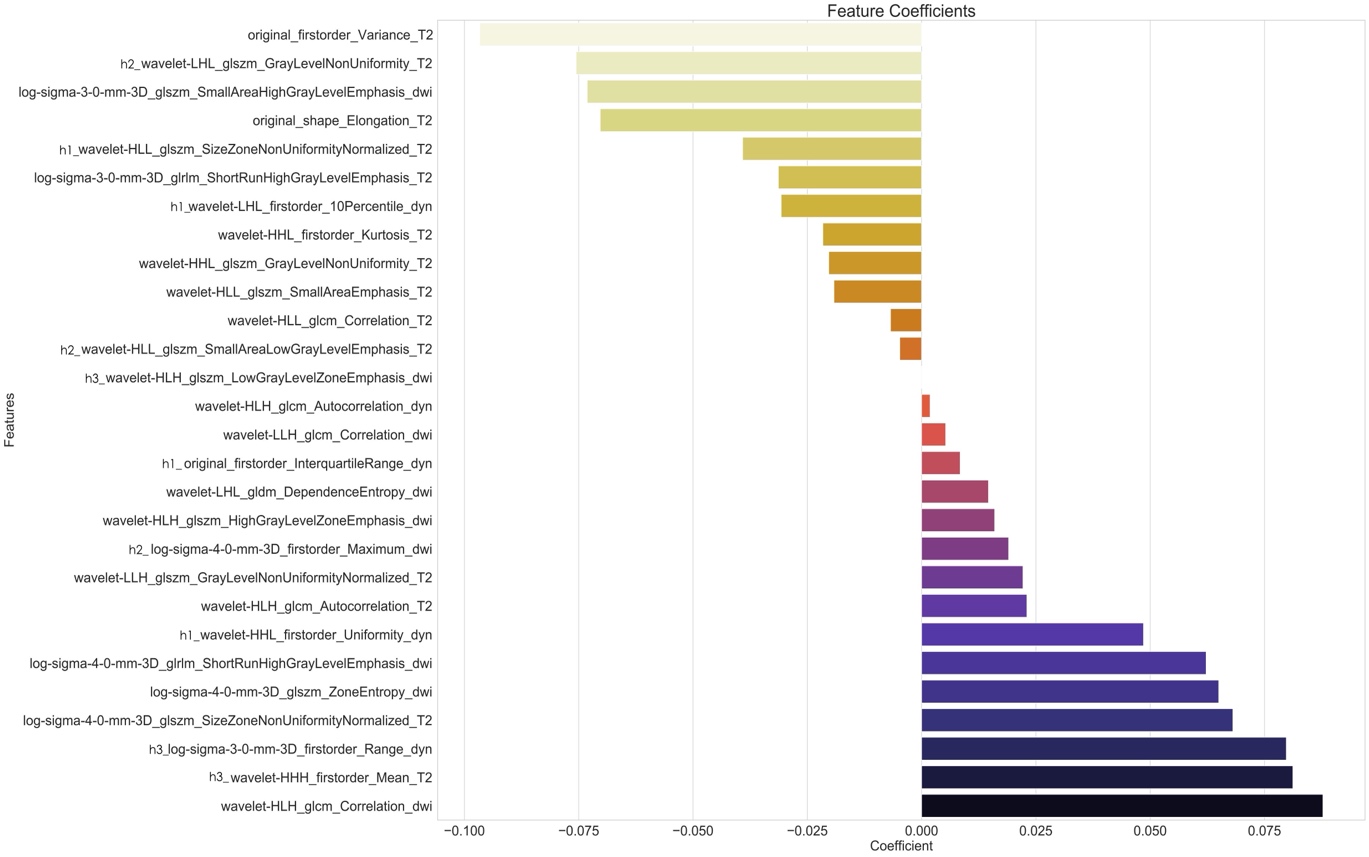

Figue S5-4: Bar plot of the non-zero radiomics feature coefficients retained in the final LASSO model, ranked by their coefficient magnitude, reflecting their relative contribution to the predictive model.

Result: Table S5-5 After LASSO retained features

Table S5-5 After LASSO retained features

| Feature group | Totall | ICC>0.90 Retained | *T* or *U* teat < 0.05 Retained | Spearman \|*ρ*\| > 0.8 Retained | mRMR Retained | LASSO Retained |
| --- | --- | --- | --- | --- | --- | --- |
| Whole tumor | 3111 | 2489 | 198 | 97 | 31 | 18 |
| Habitat | 9333 | 6066 | 315 | 76 | 19 | 10 |
| Overall | 12444 | 8555 | 513 | 173 | 50 | 28 |

Supplemental Material Table S5-6: Radiomics features and their LASSO coefficients

| number | Features | Coefficients |
| --- | --- | --- |
| 1 | wavelet-HLH_glcm_Correlation_dwi | +0.0875647 |
| 2 | wavelet-HHH_firstorder_Mean_T2_habitat3 | +0.0801531 |
| 3 | log-sigma-3-0-mm-3D_firstorder_Range_dyn_habitat3 | +0.0782312 |
| 4 | log-sigma-4-0-mm-3D_glszm_SizeZoneNonUniformityNormalized_T2WI | +0.0684850 |
| 5 | log-sigma-4-0-mm-3D_glszm_ZoneEntropy_dwi | +0.0665246 |
| 6 | log-sigma-4-0-mm-3D_glrlm_ShortRunHighGrayLevelEmphasis_dwi | +0.0625365 |
| 7 | wavelet-HHL_firstorder_Uniformity_dyn_habitat1 | +0.0496749 |
| 8 | wavelet-HLH_glcm_Autocorrelation_T2WI | +0.0187517 |
| 9 | wavelet-LLH_glszm_GrayLevelNonUniformityNormalized_T2 | +0.0187025 |
| 10 | log-sigma-4-0-mm-3D_firstorder_Maximum_dwi_habitat2 | +0.0186060 |
| 11 | wavelet-HLH_glszm_HighGrayLevelZoneEmphasis_dwi | +0.0136036 |
| 12 | wavelet-LHL_gldm_DependenceEntropy_dwi | +0.0125136 |
| 13 | original_firstorder_InterquartileRange_dyn_habitat1 | +0.0031257 |
| 14 | wavelet-LLH_glcm_Correlation_dwi | +0.0022049 |
| 15 | wavelet-HLH_glcm_Autocorrelation_dyn | +0.0017134 |
| 16 | wavelet-HLH_glszm_LowGrayLevelZoneEmphasis_dwi_habitat3 | +0.0000015 |
| 17 | wavelet-HLL_glszm_SmallAreaLowGrayLevelEmphasis_T2WI_habitat2 | -0.0052543 |
| 18 | wavelet-HLL_glcm_Correlation_T2WI | -0.0010436 |
| 19 | wavelet-HLL_glszm_SmallAreaEmphasis_T2WI | -0.0187549 |
| 20 | wavelet-HHL_glszm_GrayLevelNonUniformity_T2WI | -0.0198005 |
| 21 | wavelet-HHL_firstorder_Kurtosis_T2WI | -0.0255076 |
| 22 | wavelet-LHL_firstorder_10Percentile_dyn_habitat1 | -0.0332193 |
| 23 | log-sigma-3-0-mm-3D_glrlm_ShortRunHighGrayLevelEmphasis_T2WI | -0.0356375 |
| 24 | wavelet-HLL_glszm_SizeZoneNonUniformityNormalized_T2WI_habitat1 | -0.0390261 |
| 25 | original_shape_Elongation_T2WI | -0.0702470 |
| 26 | log-sigma-3-0-mm-3D_glszm_SmallAreaHighGrayLevelEmphasis_DWI | -0.0729357 |
| 27 | wavelet-LHL_glszm_GrayLevelNonUniformity_T2WI_habitat2 | -0.0755356 |
| 28 | original_firstorder_Variance_T2WI | -0.0949358 |

**7. Calculation of the Radiomics Score (Rad_ score)**

The final radiomics signature was constructed by a linear combination of the selected optimal features weighted by their respective non-zero coefficients derived from the LASSO regression model. For each patient, the Radiomics Score (Rad_score) was calculated using the following formula:

$$Rad-score=\beta_{0}+\sum_{i=1}^{n} \beta_{i}\chi_{i}$$

Where $\beta_{0}$ represents the intercept, $\beta_{i}$ represents the non-zero LASSO coefficient for the *i*-th selected feature, and $\chi_{i}$ represents the Z-score standardized value of the corresponding radiomics feature.

The Rad-scores of all patients were calculated using the glmnet package in R, with P-values less than 0.05 in both the training set (Figue 5-5): and test sets (Figue 5-6), indicating statistical significance. However, considering the interpretability of model features, this study did not use Rad_score. Only provided preliminary clinical imaging evaluation methods.

**8. Data Partitioning and Standardization**

To strictly prevent data leakage and avoid overfitting, the entire feature selection and model training pipeline was conducted exclusively on the training cohort. Prior to feature selection, Z-score standardization was applied to normalize the radiomics features. Specifically, the mean ($\mu$) and standard deviation ($\sigma$) of each feature were calculated solely from the training cohort. These exact training-derived parameters were subsequently applied to scale the features in the independent testing cohort. The test set also retains only these 28 features, calculated using the parameters from the training set.


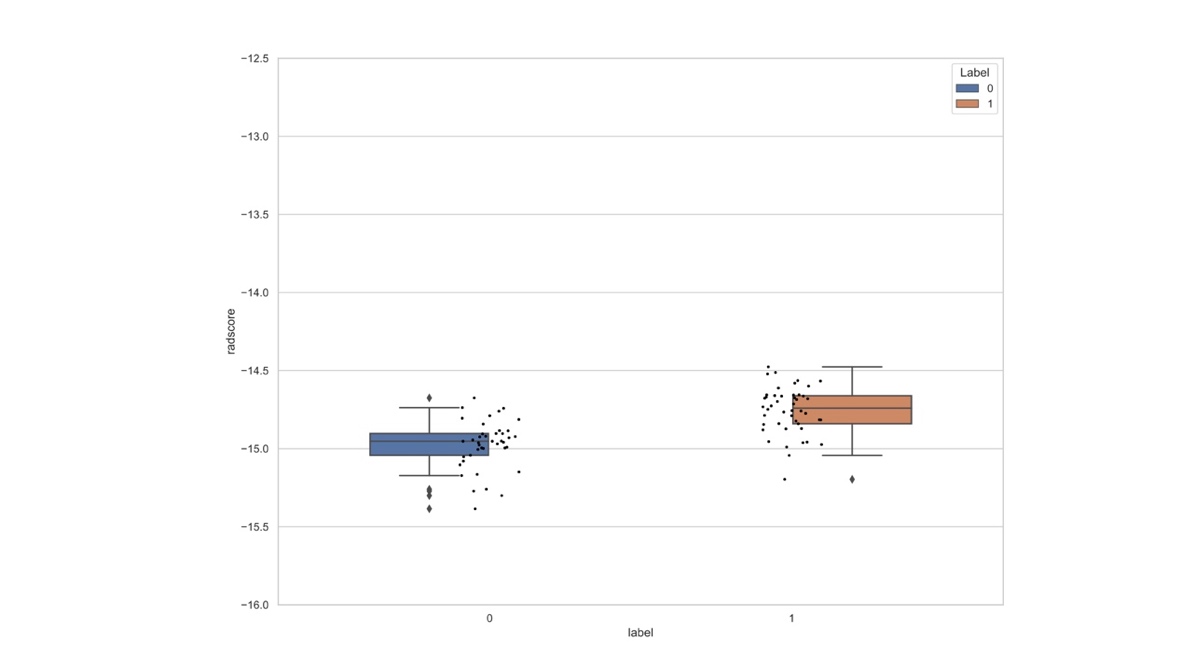


Figue S5-5: Training set 87 patients (0: non-ALNM41; 1: ALNM46)


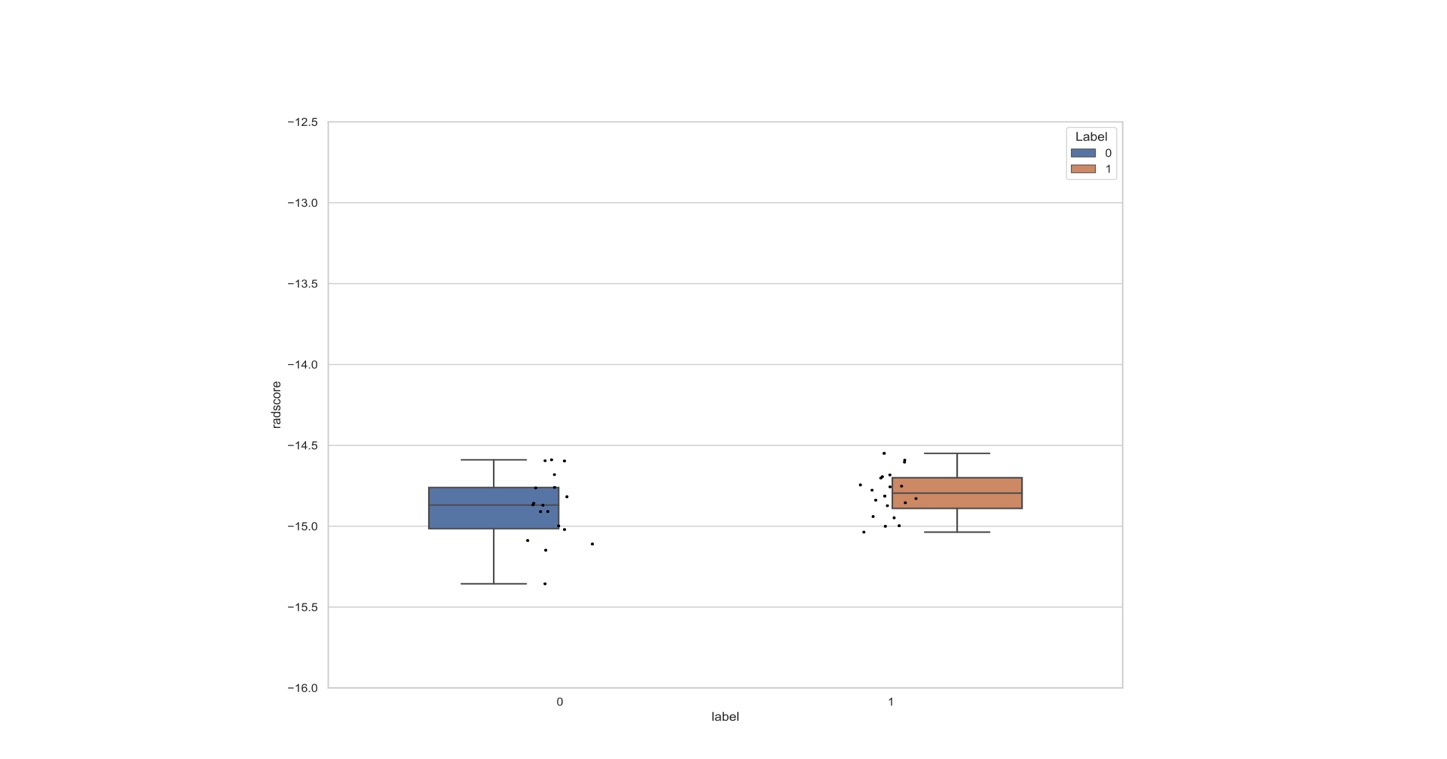


Figue S5-6: Test set 38 patients (0: non-ALNM18; 1: ALNM20)

**9. Screening results of whole tumor radiomics features and habitat radiomics features separately**

To evaluate the independent predictive value of whole-tumor and habitat features, we performed feature selection on the whole-tumor feature set, and the habitat feature set separately using the same method and subsequently constructed the models. The final selected features are shown in Figure S5 -7 and Figure S5-8. Afterwards, the two types of features were separately standardized using Z-score normalization.


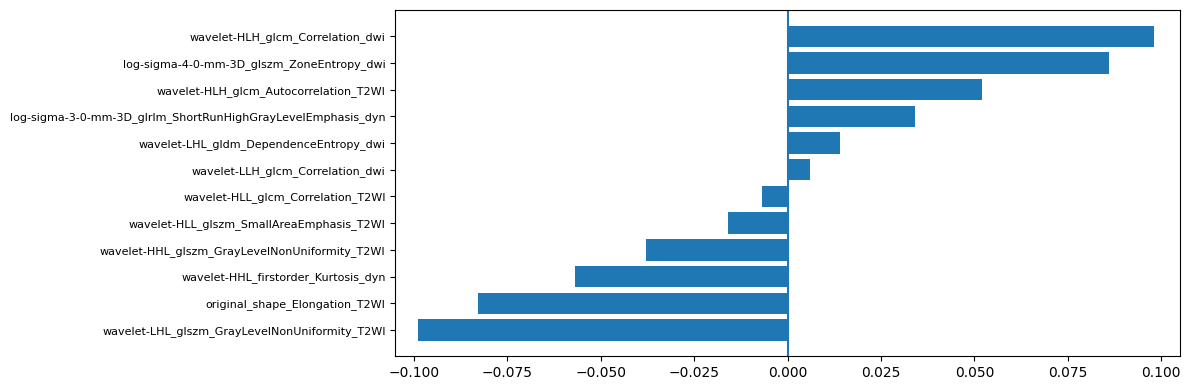


Figure S5-7: Whole tumor radiomics features (12)


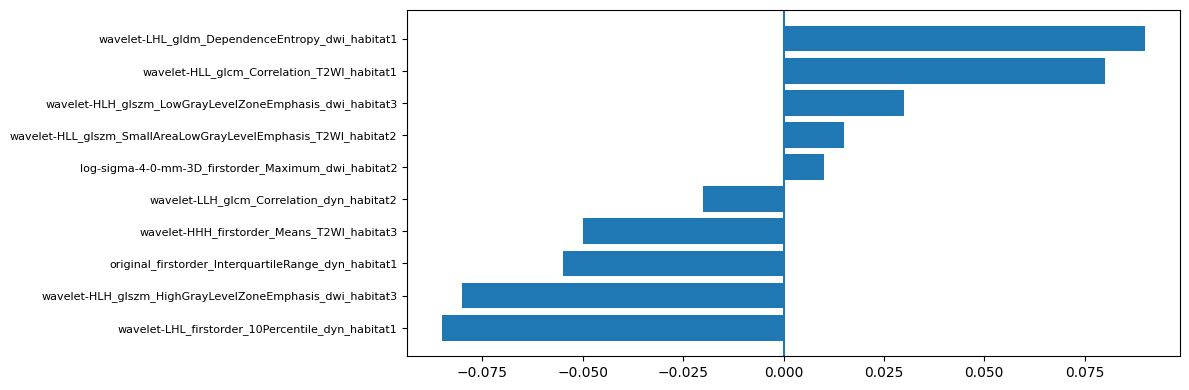
Figure S5-8: habitat radiomics features (10)

Table S5-7 Feature Distribution Table

| Model | T2WI | DWI | DCE | Total |
| --- | --- | --- | --- | --- |
| Combined model | 14 | 9 | 5 | 28 |
| Habitat radiomics model | 3 | 4 | 3 | 10 |
| Conventional radiomics | 6 | 4 | 2 | 12 |

**Supplemental Material S6.** **Finding the optimal hyperparameter： cross-validation (CV)**


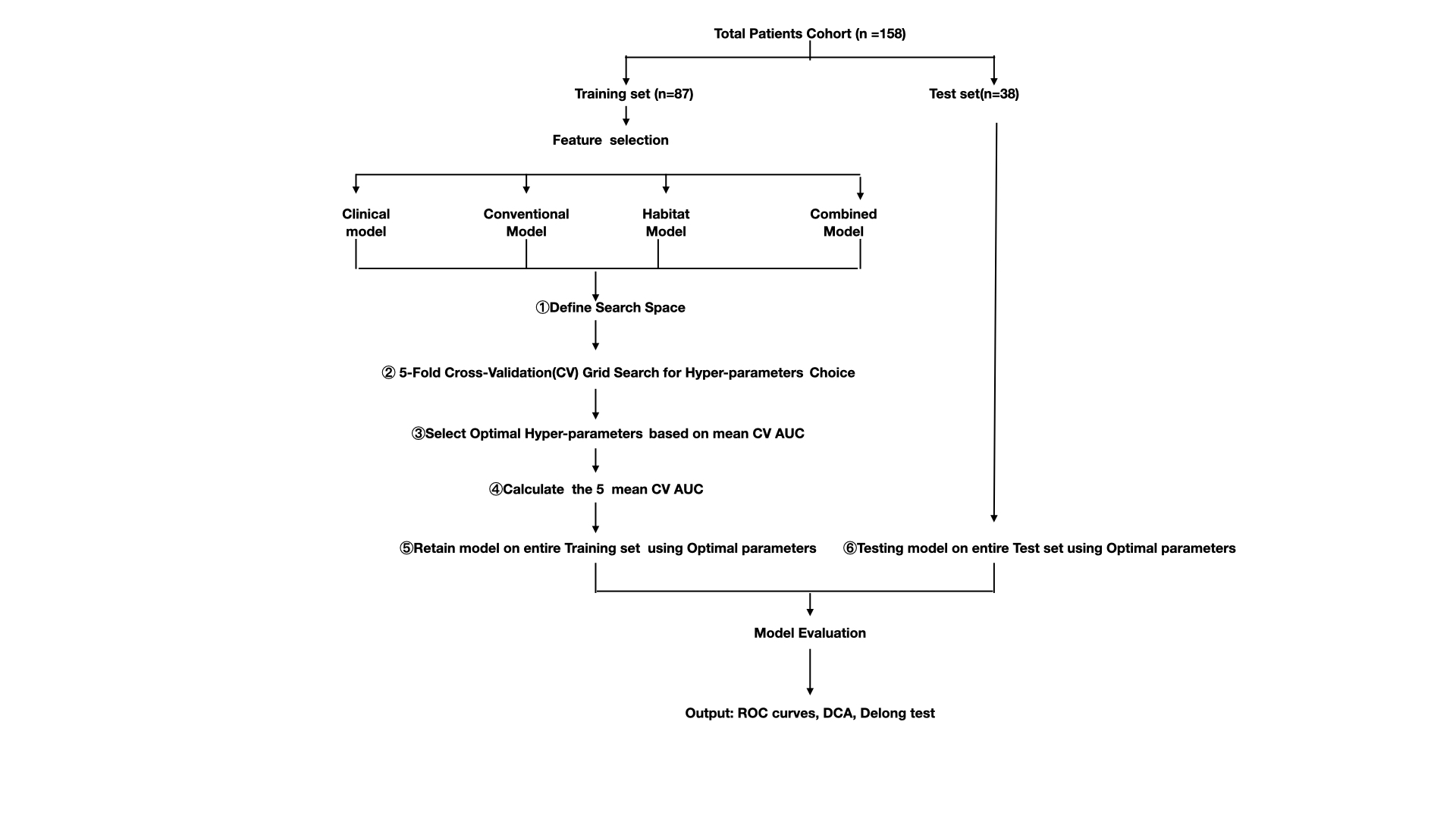


Figure S6-1 Model construction and Verification

**1.** **Introduction**

XGBoost classifiers were constructed to predict axillary lymph node metastasis (ALNM) in a supervised learning framework. Four models were developed, including the clinicopathologic model (n = 3), conventional radiomics model (n = 12), habitat radiomics model (n = 10), and combined model. The combined model incorporated 18 conventional radiomics features, 10 habitat radiomics features, and 3 independently significant clinicopathologic variables. The dataset was randomly divided into a training cohort and a validation cohort using stratified sampling to preserve the proportion of ALNM-positive cases. Model training and hyperparameter tuning were performed exclusively in the training cohort. This section mainly introduces Figure S6-1①-④ hyperparameter optimization.

**2. Hyperparameter optimization**

Hyperparameters of the XGBoost model were optimized using 5-fold stratified cross-validation within the training cohort. The training data were partitioned into 5 subsets; in each iteration, 4 subsets were used for training and the remaining subset for validation. Hyperparameter tuning was performed within each training fold using grid search combined with cross-validation, where the mean AUC across the internal validation folds was used as the criterion for selecting the optimal hyperparameter combination. The final model was configured using the optimal hyperparameters identified during cross-validation and refitted on the entire training set.

**The specific workflow is illustrated in Figure S6-1.** A cross-validation (CV) framework was employed to robustly tune hyperparameters and evaluate model performance while strictly avoiding data leakage. The detailed process is as follows:

1. Define search space: Different algorithms have strict requirements on the type and range of parameters, as detailed in Table S6-1.

**2. Hyperparameter Optimization:** Within each fold of the loop, Grid search was utilized to evaluate various hyperparameter combinations. the 5 folds. For each predefined hyperparameter combination within the search space, the model was trained and evaluated 5 times. In each iteration, 4-folds (n = 72) were utilized as the internal training subset, while the remaining 1-fold (n = 18) served as the internal validation subset. The performance of each combination was measured by calculating its validation subset (n = 18) mean Area Under the Curve (AUC) across.

**3. Optimal Parameter Selection:** The hyperparameter combination that yielded the highest mean AUC in the loop was selected as the optimal parameter set and subsequently passed to the whole training set, as detailed in Table S6-1.

**4. Cross-Validation Evaluation:** The 5-fold CV to evaluate the model's internal stability. Using the optimal parameters determined by Grid search, the model was evaluated on each respective validation fold. The mean AUC across the 5 CV AUC was calculated to assess the model's generalization ability and stability on the internal data.

**5. Model Construction (Apparent Training AUC):** Following the cross-validation, a final model was trained on the entire training dataset (n = 87) using the selected optimal hyperparameters, which represents the model's degree of fit to the known data.

**6. Independent Test Evaluation (Test AUC):**ly, the fully trained model was applied to the independent test set **(n = 38)**. The resulting Test AUC represents the model's true predictive performance and actual generalizability to unseen data.

Table 6-1. Hyperparameter search space and optimal values for the XGBoost model.

| Hyperparameter | Description | Search Grid | Optimal Value |
| --- | --- | --- | --- |
| n_estimators | Number of gradient boosted trees | [50, 100, 150, 200, 300] | 200 |
| learning_rate | Step size shrinkage used in update | [0.01, 0.05, 0.1, 0.2] | 0.05 |
| max_depth | Maximum depth of a tree | [3, 4, 5, 6, 7] | 5 |
| subsample | Subsample ratio of the training instances | [0.6, 0.7, 0.8, 0.9, 1.0] | 0.8 |
| colsample_bytree | Subsample ratio of columns when constructing each tree | [0.6, 0.7, 0.8, 0.9, 1.0] | 0.7 |
| gamma | Minimum loss reduction required to make a further partition | [0, 0.1, 0.2, 0.5, 1.0] | 0.1 |
| reg_alpha | L1 regularization term on weights | [0, 0.01, 0.1, 1.0] | 0.01 |
| reg_lambda | L2 regularization term on weights | [0.1, 1.0, 5.0, 10.0] | 1.0 |

**Note:** The optimal values were determined based on the highest mean Area Under the Curve (AUC) achieved during the -fold cross-validation within the training set on the validation set.

**Supplemental Material S7. Final model training**


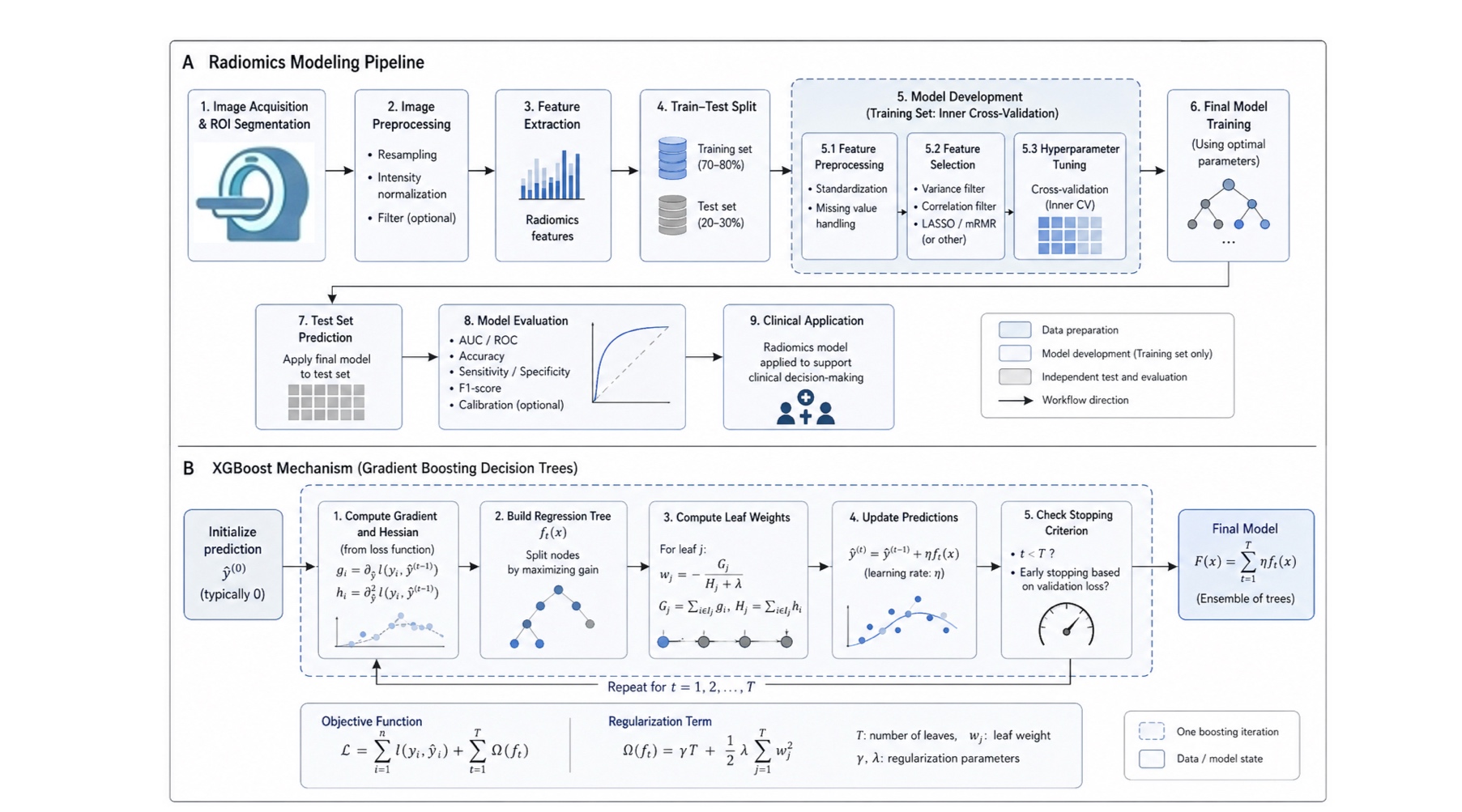


Figure S7-1 Workflow of XGBoost Model Development

**1. Introduction**

Extreme Gradient Boosting **(XGBoost)** is an efficient and regularized implementation of the gradient boosting decision tree framework. It constructs an ensemble of classification trees in a **stage-wise additive manner**, where each newly added tree is trained to correct the prediction errors made by the current ensemble. Unlike a conventional single decision tree, XGBoost aggregates the outputs of many weak trees to form a strong learner with improved predictive performance, robustness, and generalization ability. The clear workflow is shown in Part B of Figure S7-1.

**2. Principle**

2.1 Additive tree ensemble

XGBoost models the prediction as the sum of multiple decision trees:

$${y_{i}}^{\wedge}=\sum_{t=1}^{T} f_{t}\left( x_{i} \right)$$

where: $x_{i}$ denotes the feature vector of patient *I;* $f_{t}$denotes thet *t-*h regression tree; T is the number of trees in the ensemble; ${y_{i}}^{\wedge}$is the raw prediction score.

For binary classification, the raw score is converted into a probability using the sigmoid function:

$P(y_{i}=1\mid x_{i}$)$=\sigma\left( y_{i} \right)=\frac{1}{1+e^{-{y_{i}}^{\wedge}}}$

In this study, $y_{i}=1$ indicates the presence of **axillary lymph node metastasis (ALNM)**, and $y_{i}=0$indicates the absence of ALNM. Thus, the XGBoost model estimates the probability of ALNM for each patient by summing the outputs of sequentially trained trees.

2.2 Boosting mechanism

The core idea of boosting is that trees are not trained independently. Instead, they are added **sequentially**, and each new tree is trained to improve the errors of the current model. The **first tree** captures the most prominent predictive patterns in the 31-dimensional feature space. The **second tree** focuses more on samples that remain poorly predicted after the first tree. The **third tree** corrects errors left by the first two trees. This process continues until the specified number of trees is reached. Therefore, XGBoost can be understood as an iterative error-correction process, in which each tree contributes only part of the final prediction and progressively refines the model.

Inside the computer, boosting is an iterative ensemble learning method in which multiple weak or base learners are trained sequentially. The first learner is fitted to the original training data, and each subsequent learner focuses more on the samples that were misclassified or poorly predicted by the previous learners. This is typically achieved by increasing the weights of hard examples or by fitting the new learner to the residual errors. The predictions from all learners are then combined, usually through a weighted sum, to form a final strong model with improved overall accuracy and generalization performance


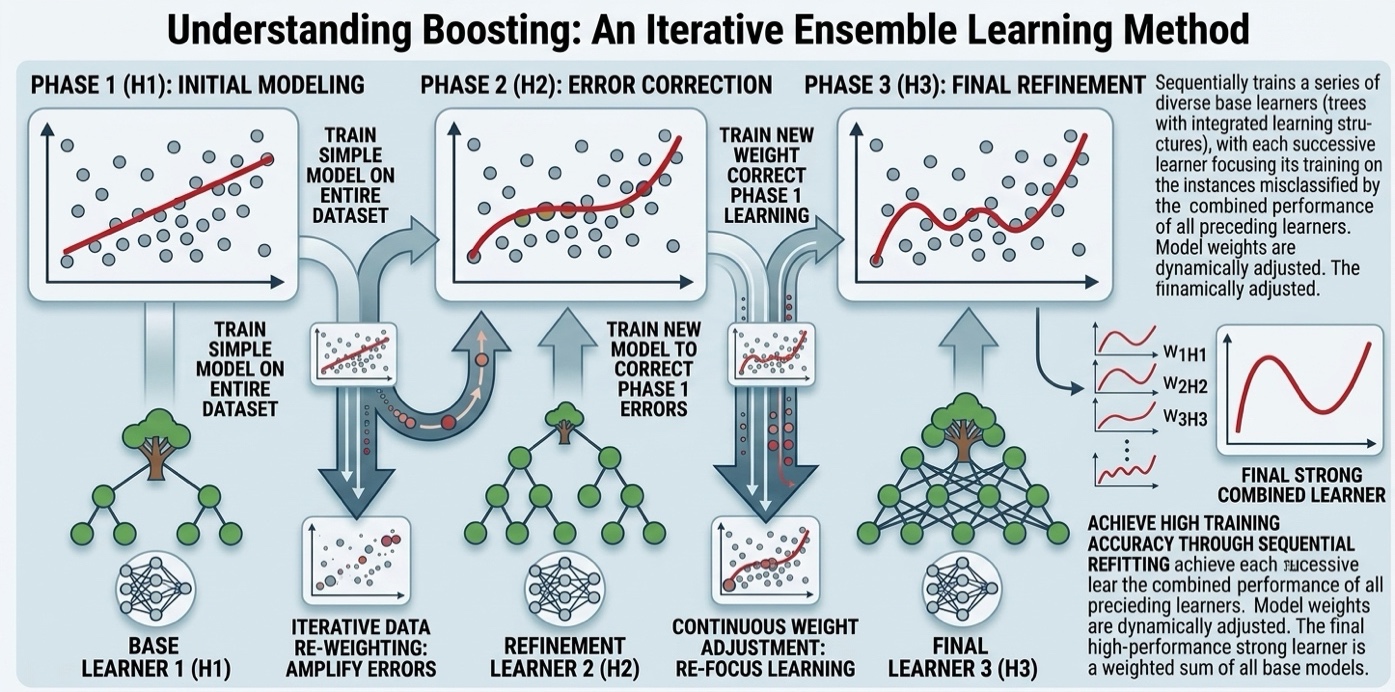


Figure S7-2 Boost algorithm. Boosting is a stage-wise ensemble method that converts multiple weak learners into a strong learner through sequential training.

**How boosting works.**

Boosting builds a strong predictive model by combining a series of relatively simple learners trained in sequence. Unlike bagging, where models are trained independently, boosting makes each new learner depend on the errors of the previous ones.

In **Phase 1**, an initial base learner (H1) is trained on the full dataset. Because this learner is simple, it may fail to capture all patterns in the data, leading to prediction errors. In **Phase 2**, the algorithm identifies the instances that were predicted incorrectly or insufficiently by H1. These difficult samples are given greater importance, either by assigning them larger weights or by defining residual errors as the new learning target. A second learner (H2) is then trained to correct these mistakes. In **Phase 3**, the process is repeated: the training emphasis is shifted toward the remaining errors after combining the earlier learners, and another learner (H3) is trained to further refine the predictions. Through this sequential error-correction strategy, each learner contributes complementary information. Finally, all learners are aggregated into a **strong combined learner**, often using a weighted combination in which better-performing learners receive larger influence. In this way, boosting progressively reduces bias and improves predictive performance, often achieving high accuracy even when each individual base learner is relatively weak.

**3. Objective Function of XGBoost**

The XGBoost model optimizes the following objective at iteration *t*:

$${Obj}^{(t)}=\sum_{i=1}^{n} \mathcal{I}\left\{ y_{i},{{y_{i}}^{\wedge}}^{(t-1)}+f_{t}(x_{i}) \right\}+\Omega(f_{t})$$

where:

$\mathcal{I}$ is the loss function

${{y_{i}}^{\wedge}}^{(t-1)}$is the prediction from the existing ensemble before adding the new tree

$f_{t}(x_{i})$is the output of the newly added tree

$\Omega(f_{t})$is the regularization term controlling tree complexity

For binary classification, the loss function is typically the **logistic loss**.

The regularization term is defined as:

$$\Omega\left( f \right)=\gamma T+\frac{1}{2}\lambda\sum_{j=1}^{T} \omega_{j}^{2}+\alpha\sum_{j=1}^{T} \mid\omega_{j}\mid$$

where:

T is the number of leaves in the tree

$\omega_{j}$is the score assigned to leaf *j*

$\gamma$penalizes additional splits

$\lambda$ is the L2 regularization coefficient

$\alpha$ is the L1 regularization coefficient

In the present study: gamma = 0.1; reg_lambda = 1.0; reg_alpha = 0.01

These terms reduce the risk of overfitting and improve generalization in a relatively small training cohort.

**4. How a Tree is Built in XGBoost**

4.1. Candidate split evaluation

At each node, XGBoost evaluates whether the current sample set should be split further. To do this, it examines: A subset of candidate features; Multiple candidate thresholds for each feature; The corresponding reduction in the objective function after splitting.

Unlike ID3 or C4.5, XGBoost does not directly use entropy or information gain. Unlike a standard CART classifier, it does not directly use the Gini index as the split criterion. Instead, it uses a gain function derived from first- and second-order derivatives of the loss function.

4.2. Gradient and Hessian

For each sample *i*, XGBoost computes:

the first-order gradient:

$$g_{i}=\frac{\partial l(y_{i,}y_{i}^{\wedge})}{\partial y_{i}^{\wedge}}$$

the **second-order gradient (Hessian)**:

$$h_{i}=\frac{\partial^{2}l(y_{i,}y_{i}^{\wedge})}{\partial{y_{i}^{\wedge}}^{2}}$$

These quantities describe: The direction in which the prediction should be adjusted. The curvature of the loss around the current prediction. New tree is trained to fit these gradient statistics rather than directly fitting the original labels.

4.3. Split gain

Suppose a node is split into a left child and a right child. Let:

$G_{L}=\sum_{i\epsilon I_{L}} g_{i}$ $H_{L}=\sum_{i\epsilon I_{L}} h_{i}$

$G_{R}=\sum_{i\epsilon I_{R}} g_{i}$ $H_{R}=\sum_{i\epsilon I_{R}} h_{i}$

G=$G_{L}+G_{R}$ H=$H_{L}+H_{R}$

The gain produced by this split is:

Gain=$\frac{1}{2}(\frac{G_{L}^{2}}{\left( H_{L} \right)+\lambda}+\frac{G_{R}^{2}}{\left( H_{R} \right)+\lambda}-\frac{G^{2}}{H-R}$)-$\gamma$

where:

$\lambda$ corresponds to reg_lambda

$\gamma$ corresponds to the minimum loss reduction required for an additional split

A split is accepted only if it yields a sufficiently positive gain.

Therefore, XGBoost selects the feature and threshold that maximize the reduction in the regularized objective function.

**5.** **Interpretation of Tree Splitting in This Study**

In combined model, each patient is represented by a vector of 31 predictors: 28 radiomics features, including first-order, shape, and texture descriptors extracted from whole-tumor and habitat subregions across multiparametric MRI sequences, a root node in one tree may evaluate candidate splits such as: ALN-length ≤ 1.8 cm; Grade ≥ 3; wavelet-HLH_glcm_Correlation_dwi ≤ 0.42; original_firstorder_Variance_T2WI ≤ -0.31.

The algorithm computes the gain associated with each candidate split and selects the one that most improves the model fit.

If the split with the highest gain: a root node in one tree may evaluate candidate splits such as: ALN-length ≤ 1.8 cm; Grade ≥ 3; wavelet-HLH_glcm_Correlation_dwi ≤ 0.42; original_firstorder_Variance_T2WI ≤ -0.31.

The algorithm computes the gain associated with each candidate split and selects the one that most improves the model fit. If the split with the highest gain is: ALN-length≤1.8. Then the node is divided accordingly: left branch: patients with shorter ALN-length; right branch: patients with longer ALN-length.

Subsequent splits may then involve TIC, Grade, or any radiomics feature, depending on which variable best partitions the residual error pattern in that node. Thus, each decision path can be interpreted as a. **hierarchical combination of clinicopathologic and radiomic conditions** associated with a specific ALNM risk increment.

**6. Leaf Weight Assignment**

Once the structure of a tree is determined, each terminal node (leaf) is assigned an output score:

$$\mathcal{W}_{j}^{*}=-\frac{G_{j}}{H_{j}+\lambda}$$

where:

$G_{j}$is the sum of gradients in leaf *j*

$H_{j}$is the sum of Hessians in leaf *j*

$\lambda$ is the L2 regularization coefficient

This leaf score represents how much the prediction should be adjusted for all samples falling into that leaf. Interpretively: ***positive leaf weight*** increases the predicted probability of ALNM; ***negative leaf weight*** decreases it. Each patient receives the corresponding leaf score from each tree, and the scores from all trees are added together.

**7. Each Tree Corrects the Errors of the Previous Trees**

After the first tree is built, each patient obtains an initial prediction. However, some patients remain underpredicted or overpredicted. After the first tree is built, each patient obtains an initial prediction. However, some patients remain underpredicted or overpredicted. The next tree is then trained to focus more strongly on these remaining errors. Importantly, the new tree does not directly refit the original label; instead, it fits the **gradient and Hessian information** generated by the current predictions. Therefore, the newly added tree specifically targets the residual structure left unexplained by the preceding ensemble. This process is repeated iteratively across *n_estimators = 200* trees, so that the final model becomes a refined additive combination of many small correction steps.

**8. Role of the Learning Rate**

The contribution of each newly added tree is scaled by the learning rate:

$$y_{i}^{\wedge(t)}=y_{i}^{\wedge(t-1)}+\eta f_{t}(x_{i})$$

where $\eta$ is the learning rate. In this study: $\eta=0.05$. This means that each tree makes only a small update to the current prediction. Such shrinkage has two major advantages: It stabilizes the learning process. It reduces overfitting by preventing large, abrupt corrections. Because each tree contributes only modestly, more trees are needed, which explains the selection of *n_estimators = 200*.

**9.** **Pre-pruning and Complexity Control**

XGBoost incorporates pre-pruning by restricting tree growth during the training process, rather than growing a fully expanded tree first and then pruning it afterward.

In this study, pre-pruning was mainly achieved through the following hyperparameters:

**(1) max_depth *=* 5** Each tree was limited to a maximum depth of 5. This means that a single decision path can contain at most five split decisions. This constrains the complexity of each weak learner and prevents overly deep trees from memorizing noise.

**(2) gamma = 0.1** A split was accepted only if it reduced the objective function by at least 0.1. This acts as a minimum gain threshold and prevents uninformative or marginal splits from being added.

**(3) subsample = 0.8** Each tree was trained on a random 80% subsample of the training cohort. This reduces variance and enhances robustness, especially in relatively small datasets.

**(4) colsample_bytree = 0.7** Each tree used only 70% of the available predictors. Given the multimodal and potentially correlated nature of radiomics variables, this feature subsampling helps reduce dominance by a small subset of variables and improves ensemble diversity.

**(5) reg_alpha = 0.01 and reg_lambda = 1.0** These L1 and L2 regularization terms penalize large or unstable leaf weights and further constrain the model. Together, these mechanisms act as a multi-level complexity control framework.

Thus, the final hyperparameters represent the empirically best-performing trade-off between model flexibility and generalization within the training cohort.

**10. Calculate probability**

Finally, the trees are added to the set to obtain a score, which is then transformed into a predicted probability via the sigmoid function. The sigmoid function is used to convert the model’s raw output (a score that can range from negative to positive infinity) into a probability between 0 and 1. Mathematically, it is defined as:

$$y=\frac{1}{1+e^{-x}}$$

**Supplemental Material S8 Model Evaluation**

**1.Prediction and ROC analysis**

1.1. Principle

The Receiver Operating Characteristic (ROC) curve is used to evaluate the discriminative performance of a predictive model across all possible classification thresholds. A series of thresholds is applied to these predicted probabilities. For each threshold: Patients with predicted probability above the threshold are classified as positive. Patients below the threshold are classified as negative. At each threshold, two quantities are calculated: **True Positive Rate (TPR, sensitivity):** proportion of actual positive cases correctly identified. **False Positive Rate (FPR, 1 − specificity):** proportion of actual negative cases incorrectly classified as positive. By continuously varying the threshold from the highest to the lowest predicted probability, a sequence of (FPR, TPR) pairs is obtained. Plotting these points produces the ROC curve.

The ROC curve reflects how well the model separates positive and negative cases based on its predicted scores. Conceptually, it corresponds to scanning down the ranked list of patients and progressively including more individuals as positive. A model with good discriminative ability will assign higher probabilities to true positive cases than to negative ones. This results in a curve that rises quickly toward the upper-left corner. In contrast, a model with poor discrimination produces a curve closer to the diagonal line, indicating random performance. The Area Under the Curve (AUC) summarizes the ROC curve into a single value. It can be interpreted as the probability that a randomly selected positive case is assigned a higher predicted probability than a randomly selected negative case.

1.2. Specific methods

The trained XGBoost model outputs a predicted probability of ALNM for each patient. Receiver operating characteristic (ROC) curves were generated by plotting the true positive rate (sensitivity) against the false positive rate (1 − specificity) at various probability thresholds using the *scikit-learn* function *roc_curve*. The area under the ROC curve (AUC) was calculated using the *auc* function to quantify model discrimination performance. ROC analysis was performed separately in the training and validation cohorts.

**2. Delong test**

2.1. Principle

The DeLong test is a nonparametric method used to statistically compare the areas under two correlated receiver operating characteristic curves (AUCs), particularly when both models are evaluated on the same dataset.

2.2. AUC

The AUC can be interpreted as the probability that a randomly selected positive sample is assigned a higher score than a randomly selected negative sample. It can be expressed as the average of pairwise comparisons:

$$AUC=\mathbb{E[\emptyset}\left( X,Y \right)]$$

where:

$$\emptyset\left( X,Y \right)=\left\{ \begin{aligned} 1, X>Y \\ 0.5, X=Y \\ 0, X<Y \end{aligned} \right.$$

2.3. Estimation of Variance and Covariance.

DeLong’s method decomposes the AUC into contributions from individual samples: (1) $V_{i}$: average comparison outcome of the *i*-th positive sample against all negative samples. (2) $W_{j}$: average comparison outcome of the *j*-th negative sample against all positive samples.

These quantities are used to estimate Variance of each AUC; Covariance between two AUCs (since they are computed on the same dataset)

4.4. Z Statistic

The difference between two AUCs is standardized using the estimated variance:

$$Z=\frac{{AUC}_{1}-{AUC}_{2}}{\sqrt{\left( Var({AUC}_{1} \right)+\left( Var({AUC}_{2} \right)-2Cov\left( {AUC}_{1},{AUC}_{12} \right)}}$$

where:

AUC1, AUC2: areas under the ROC curves of two models

Var: variance estimated via DeLong’s method

Cov: covariance accounting for paired data

2.5. Conversion from Z Value to P Value

Under the null hypothesis (no difference between AUCs), the Z statistic approximately follows a standard normal distribution:

$$Z\sim N(0,1)$$

The two-sided p-value is calculated as:

$$P=2\left[ 1-\Phi\left( \mid Z\mid\right) \right]$$

where $\Phi$is the cumulative distribution function of the standard normal distribution.

A large absolute Z value indicates that the difference between AUCs is large relative to its variability

A small absolute Z value indicates that the difference may be due to random variation

Accordingly:

Large |Z| → small p-value → statistically significant difference

Small |Z| → large p-value → no statistically significant difference

2.6 Specific methods

All analyses were performed in Python. AUC values were calculated using the *scikit-learn* package (*sklearn.metrics.roc_auc_score*). The statistical comparison between two correlated ROC curves was conducted using the DeLong test implemented in Python, based on the *numpy* and *scipy* libraries.

Specifically, the predicted probabilities of the two models and the corresponding binary labels were used as input. The Z statistic was computed based on the difference between the two AUCs and their estimated variance–covariance structure, and the corresponding two-sided p-value was derived using the standard normal distribution implemented in *scipy. stats*. A two-sided *p*-value < 0.05 was considered statistically significant.

2.7. Result

Table S8-1 Comparisons of predictive models using Delong test in the test set

| Model | Combined Model | Habitat Model | Radiomic Model | Clinical Model |
| --- | --- | --- | --- | --- |
| Combined Model | —— | 0.032^*^ | 0028^*^ | 0.040^*^ |
| Habitat Model | 0.032^*^ | —— | 0.015^*^ | 0.018^*^ |
| Conventional radiomic Model | 0.028^*^ | 0.015^*^ | —— | 0.051 |
| Clinical Model | 0.040^*^ | 0.018^*^ | 0.051 | —— |

Note: (*) representative statistical significance: *P* < 0.05

**3. Calibration assessment**

3.1. Principle

Calibration curves evaluate the agreement between predicted probabilities and observed outcome frequencies, assessing whether a model’s predicted risks are numerically accurate.

Generation Process: Step 1: Obtain Predicted Probabilities： For each patient, the model outputs a predicted probability of the outcome. Step 2: Group Patients by Predicted Risk. All patients are divided into several groups (we choise 5 bins) based on their predicted probabilities. Grouping: 0.0–0.2, 0.2–0.4, 0.4–0.6, 0.6–0.8，0.8–1.0.Patient with similar predicted risks is placed in the same group. Step 3: Summarize Each Group.For each group: Compute the average predicted probability (what the model claims). Compute the observed event rate (what happened). **Step 4: Plot the Relationship.** Each group is represented as a point: x-axis: average predicted probability; y-axis: observed event rate. Connecting these points forms the **calibration curve**. For a bin coordinate point (x, y) = (average predicted probability, actual occurrence rate)

3.2 Interpretation

Each point on the calibration curve represents a group of patients with similar predicted probabilities, where the x-coordinate corresponds to the mean predicted probability and the y-coordinate corresponds to the observed event rate within that group. The diagonal line (45° line) represents **perfect calibration**: predicted risk equals observed risk. If the curve lies: **Below the diagonal**: the model **overestimates risk. Above the diagonal**: the model **underestimates risk.**

3.3. Specific methods

Calibration curves were generated using the *calibration_curve* function from *scikit-learn*, with predicted probabilities grouped into bins and compared with the observed event rates.

**4** **Hosmer–Lemeshow test**

4.1. Principle

The Hosmer–Lemeshow (HL) goodness-of-fit test is a statistical method used to evaluate the **calibration** of a binary prediction model. Calibration refers to the agreement between the **predicted probabilities** generated by a model and the **observed event frequencies** in the data.

4.2. Study-specific grouping strategy

In this study, the dataset consisted of: Training cohort: 87 patients; Test cohort**:** 38 patients. Because the test cohort was relatively small, the HL test was performed by dividing patients into 5 groups according to predicted risk, rather than using the conventional 10-group decile approach. This grouping strategy was adopted to avoid excessively sparse groups and to improve the stability of the observed-versus-expected comparison. Thus, for both the training set and the test set:

$$G=5$$

where $G$ denotes the number of groups.

After sorting patients by their predicted probabilities from lowest to highest, they were partitioned into 5 approximately equal-sized groups.

4.3. Mathematical Formulation

Let:

$N$ denote the total number of patients in each set

$G$ denote the number of groups ($G=5$)

$y_{i}=\left\{ 0,1 \right\}$ denote the observed binary outcome for patient $i$, were

$$y_{i}=\left\{ \begin{aligned} 1, if ALNM is present \\ 0,if ALNM is absent \end{aligned} \right.$$

$P_{i}^{\wedge}\epsilon\left[ 0,1 \right]$ denote the predicted probability of ALNM for patient $i$

Patients are first ranked by $P_{i}^{\wedge}$ and divided into $G$ groups.

For the $g$-th group, define:

$n_{g}$: number of patients in group $g$

$O_{g}$: observed number of events in group $g$

$E_{g}$: expected number of events in group $g$

The observed number of events is:

$$O_{g}=\sum_{i\epsilon g} y_{i}$$

The expected number of events is:

$$E_{g}=\sum_{i\epsilon g} P_{i}^{\wedge}$$

The observed number of non-events is:

$$n_{g}-O_{g}$$

The observed number of non-events is:

$$n_{g}-E_{g}$$

The expected number of non-events is:

$$HL=\sum_{g=1}^{G} \left\{ \frac{\left( \left( O_{g}-E_{g} \right)^{2} \right)}{E_{g}}+\frac{\left[ \left( n_{g}-O_{g} \right)-\left( n_{g}-E_{g} \right) \right]^{2}}{n_{g}-E_{g}} \right\}$$

Since

$$\left( n_{g}-O_{g} \right)-\left( n_{g}-E_{g} \right)=E_{g}-O_{g}$$

the formula may also be written equivalently as:

$$X_{HL}^{2}=\sum_{g=1}^{G} \left[ \frac{\left( O_{g}-E_{g} \right)^{2}}{E_{g}}+\frac{\left( O_{g}-E_{g} \right)^{2}}{{n_{g}-E}_{g}} \right]$$

These formulations are mathematically equivalent.

4.4 Distribution of the Test Statistic and P-value

Under the null hypothesis that the model is well calibrated, the HL statistic approximately follows a chi-square distribution with:

$$df=G-2$$

Because $G=5$ in this study:

$$df=5-2=3$$

The P-value is therefore calculated as:

$$P=P(X_{3}^{2}\geq X_{HL}^{2},observed)$$

$\mathrm{where}X_{HL}^{2},observed$ is the calculated HL statistic.

4.5. Hypotheses and Interpretation

The hypotheses of the HL test are:

Null hypothesis $H_{0}$: the predicted probabilities agree with the observed event frequencies; the model is adequately calibrated.

Alternative hypothesis $H_{1}$: the predicted probabilities differ significantly from the observed event frequencies; the model is not well calibrated.

Interpretation:

*P* > 0.05 indicates no statistically significant departure from perfect calibration and suggests **adequate calibration**.

*P* < 0.05 indicates a statistically significant discrepancy between predicted and observed outcomes and suggests **poor calibration**.

Because the HL test may be influenced by sample size and grouping strategy, its results were interpreted together with the **calibration curves** rather than in isolation.

4.6. Software Implementation

The HL test was implemented in ***R*** using the ***ResourceSelection*** package (g = 5).

4.7 Result

Table S8-2 Hosmer–Lemeshow test *P*-value

| Model | Cohort | HL *P*-value |
| --- | --- | --- |
| Combined model | Training set | 0.49^*^ |
|  | Test set | 0.13^*^ |
| Habitat model | Training set | 0.23^*^ |
|  | Test set | 0.12^*^ |
| Conventional radiomic model | Training set | 0.19^*^ |
|  | Test set | 0.08^*^ |
| Clinical model | Training set | 0.09^*^ |
|  | Test set | 0.06^*^ |

Note: (*) representative *P* > 0.05 indicates no statistically significant departure from perfect calibration and suggests **adequate calibration**.

**5. Decision curve analysis (DCA)**

5.1. Principle

Decision curve analysis (DCA) was performed to evaluate the clinical utility of the models by quantifying the net benefit across a range of threshold probabilities. Net benefit was calculated according to the following formula:

$$Net Benefit=\frac{TP}{N}-\frac{FN}{N}\times\frac{P_{t}}{1-P_{t}}$$

where TP and FP denote the number of true positives and false positives, respectively, N is the total number of patients, and Pt is the threshold probability. The net benefit of each model was compared with the default strategies of treating all patients and treating none. Interpretation of Net Benefit: A higher net benefit indicates a better clinical decision strategy. The weighting factor$\frac{P_{t}}{1-P_{t}}$reflects how harmful a false positive is relative to a false negative. DCA curves were plotted to visualize clinical usefulness.

5.2 Construction of the Decision Curve

The DCA curve is generated through the following steps: Define a sequence of threshold probabilities. For each threshold: classify patients based on predicted probabilities; calculate TP and FP; compute net benefit. Plot: x-axis: threshold probability; y-axis: net benefit.

5.3. Specific methods

Decision curve analysis was implemented in Python using a combination of standard scientific computing libraries. Predicted probabilities were generated using models developed with the *scikit-learn* package. Numerical computations, including the calculation of true positives, false positives, and net benefit across threshold probabilities, were performed using *NumPy* and *pandas*.

Net benefit was calculated manually according to the standard definition across a range of threshold probabilities. For each threshold, patients were classified based on whether their predicted probability exceeded the threshold, and corresponding true positive and false positive counts were derived.

Decision curves were then visualized using *Matplotlib*, with optional refinement and aesthetic adjustments performed using *Seaborn*. As no universally adopted dedicated Python package for decision curve analysis currently exists, all computations were implemented through custom code to ensure transparency and reproducibility.

**6. Confusion Matrix (CM)**

6.1. Principle

A **confusion matrix** summarizes the performance of a binary classifier by comparing predicted labels with true (ground-truth) labels.

For a binary classification problem, the confusion matrix consists of four elements:

**True Positives (TP):** Model predicts positive and the true label is positive

**True Negatives (TN):** Model predicts negative and the true label is negative

**False Positives (FP):** Model predicts positive, but the true label is negative

**False Negatives (FN):** Model predicts negative, but the true label is positive

6.2. Constructed Method

The model outputs **predicted probabilities** $\hat{p}_{i}\in[0,1]$ for each sample $i$.

A **classification threshold** (here, 0.5) is applied:

$$y_{i}^{\wedge}=\left\{ \begin{aligned} 1, if \left( P_{i}^{\wedge}\geq0.5 \right) \\ 0, if \left( P_{i}^{\wedge} \right)<0.5 \end{aligned} \right.$$

The predicted labels $y_{i}^{\wedge}$ are compared with the ground-truth labels $y_{i}\epsilon\left\{ 0,1 \right\}$

Counts of TP, TN, FP, and FN are computed across all samples.

6.3. Mathematical Definitions of Metrics

Sensitivity (Recall, True Positive Rate)

$$Sensitivity=\frac{TP}{TP+FN}$$

Specificity (True Negative Rate)

$$Specificity=\frac{TN}{TN+FP}$$

Accuracy

$$Accuracy=\frac{TP+TN}{TP+TN+FP+FN}$$

Precision (Positive Predictive Value, PPV)

$$PPV=\frac{TP}{TP+FP}$$

Negative Predictive Value (NPV)

$$NPV=\frac{TP}{TP+FP}$$

Confusion matrices were generated using the *scikit-learn* library in Python. Sensitivity, specificity, accuracy, precision (PPV), and negative predictive value (NPV) were calculated according to their standard mathematical definitions based on the counts of true positives (TP), false positives (FP), true negatives (TN), and false negatives (FN).

6.4. **Confusion matrix** visualization


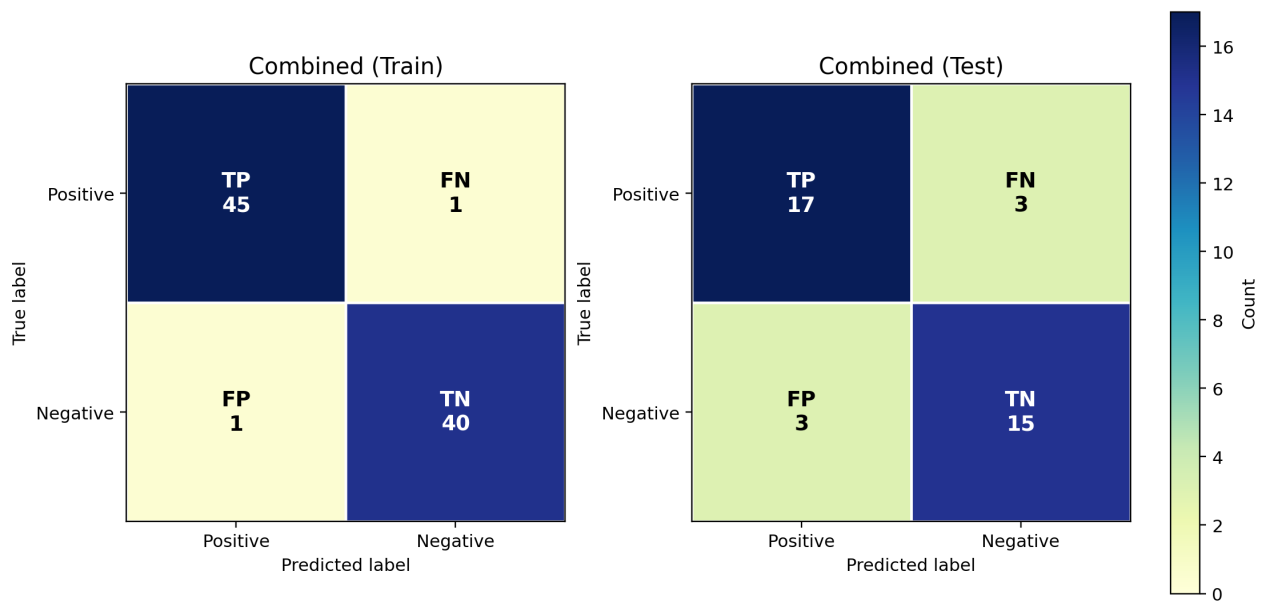


Figure 8-1 Combined model CM


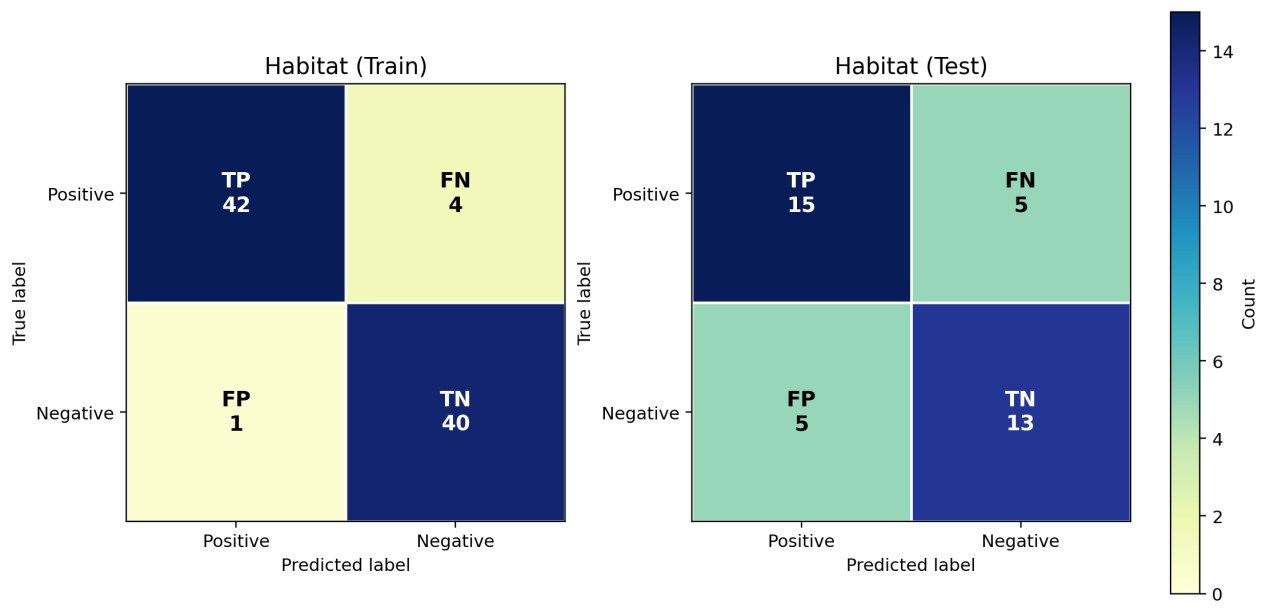


Figure S8-2 Habitat radiomics model CM


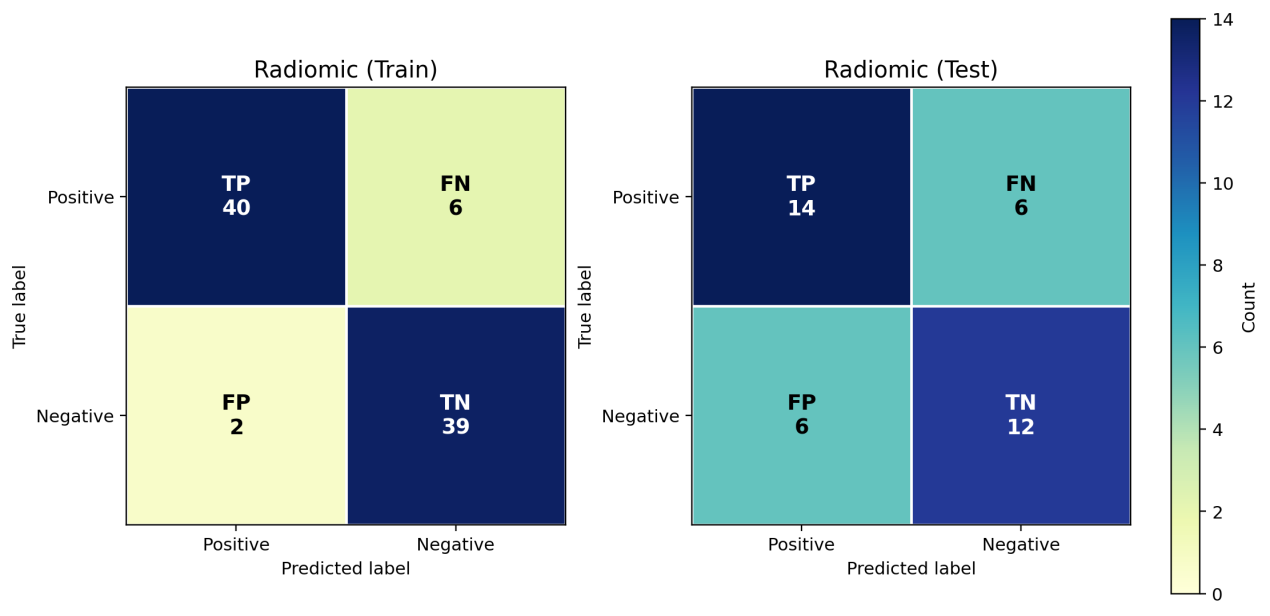


Figure 8-3 Conventional radiomics model CM


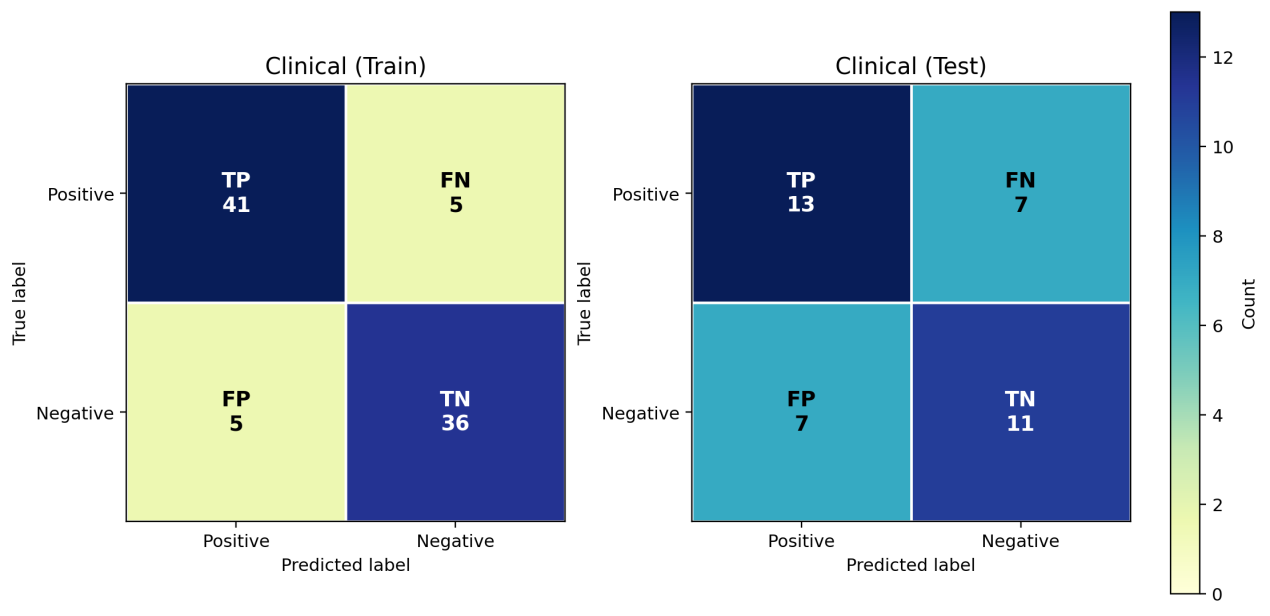


Figure 8-4 Clinical model CM

**Supplemental Material S9. Model Interpretability Analysis**

**1. Principle of SHAP**

SHAP is based on the concept of Shapley values, originally derived from cooperative game theory. In this framework, each feature is regarded as a “player” in a game, and the final model prediction is considered the “payout” generated jointly by all features. The Shapley value of a given feature represents its average marginal contribution to the prediction across all possible combinations of features.

For each patient, the model prediction can be decomposed as:

$$f\left( x \right)=\emptyset_{0}+\sum_{i=1}^{M} \emptyset_{i}$$

where *f(x)* is the model output for that patient, $\emptyset_{0}$is the baseline model output (ie, the average prediction across the dataset), *M* is the total number of features, and$\emptyset_{i}$is the SHAP value of the i-th feature. A positive SHAP value indicates that the feature increases the predicted risk of ALNM, whereas a negative SHAP value indicates that the feature decreases the predicted risk. Because XGBoost is a tree-based model, SHAP values can be computed efficiently using the ***TreeExplainer*** algorithm, which is specifically designed for tree ensemble models.

**2. Software implementation**

SHAP analysis was performed in Python using the *shap* package. After model development and hyperparameter optimization in the training cohort, the final XGBoost classifier was refit on the full training cohort. To interpret model decisions under independent evaluation conditions, SHapley Additive exPlanations (SHAP) were computed on the held-out test set (n = 38). SHAP values were calculated based on the final trained model and the test feature matrix *X_test_* without updating any model parameters. For tree-based models, we used *shap. TreeExplainer*, which provides efficient and exact/consistent Shapley-value computation for XGBoost. Global feature importance was summarized by the mean absolute SHAP value.

**3. Image explanation**

3.1. SHAP summary bar plot

The SHAP summary bar plot was used to show the global importance of all included features. It ranks features according to the mean absolute SHAP value across all patients. In other words, it reflects how strongly each feature contributes to the model predictions on average, regardless of direction. A larger mean absolute SHAP value indicates that the feature has a greater overall effect on model output. The plot does not show whether the feature increases or decreases risk; it only reflects the magnitude of contribution. Therefore, this plot is useful for identifying the most influential predictors in the final model. In the present study, the SHAP summary bar plot was used to rank conventional radiomics features, habitat radiomics features, and clinicopathologic variables according to their average absolute contribution to ALNM prediction. This analysis provided a global measure of feature importance in the final XGBoost model.

3.2. SHAP beeswarm plot

The SHAP beeswarm plot was used to illustrate both the importance and direction of feature effects across all patients. Compared with the bar plot, the beeswarm plot provides more detailed information because it displays the SHAP value of each feature for each individual patient.

In the beeswarm plot: Each dot represents one patient. The x-axis represents the SHAP value. Positive SHAP values indicate that the feature pushes the prediction toward ALNM positivity. Negative SHAP values indicate that the feature pushes the prediction toward ALNM negativity. The color indicates the actual feature value: Red represents a high feature value. Blue usually represents a low feature value. Features are ordered from top to bottom according to overall importance. This plot allows visualization of which features are most important, whether high or low values of a feature are associated with higher predicted risk, and whether the feature effect is consistent or heterogeneous across patients. The SHAP beeswarm plot was used to visualize the distribution of feature effects across the study population. For each feature, the horizontal position of each point indicates the magnitude and direction of its contribution to the model output, while color encodes the original feature value. This plot enabled identification of whether higher or lower values of a given radiomics or clinicopathologic feature were associated with increased predicted risk of ALNM.

3.3. SHAP heatmap

The SHAP heatmap was used to display the pattern of SHAP values across all patients and features simultaneously. It provides an overview of how feature contributions vary between individuals and can reveal clusters of patients with similar explanation patterns. Rows correspond to patients. Columns correspond to features. The color intensity reflects the SHAP value: warm colors often indicate positive contribution to ALNM prediction, cool colors indicate negative contribution. Patients with similar SHAP patterns may appear grouped together. This plot is particularly useful for showing heterogeneity of feature contributions across the cohort. The SHAP heatmap was generated to visualize patient-level heterogeneity in model explanations. By jointly displaying SHAP values for multiple features across all patients, the heatmap provides a compact representation of how the combined radiomics and clinicopathologic signatures contributed to ALNM prediction at the individual level.

3.4. SHAP waterfall plot

The SHAP waterfall plot was used to explain the model prediction for a single patient. It decomposes the prediction into the baseline value and the additive contributions of individual features. For one selected patient: The plot starts from the base value, which represents the average model output over the dataset. Each feature then pushes the prediction higher or lower. Features with positive SHAP values increase the predicted probability of ALNM. Features with negative SHAP values decrease the predicted probability. The final output corresponds to the predicted model score for that patient. This plot is especially useful for case-level interpretation because it shows exactly which radiomics and clinical features drove the prediction in a specific patient. The SHAP waterfall plot was used for individual-level interpretation of the final XGBoost model. For a representative patient, the predicted ALNM risk was decomposed into the baseline prediction and the additive contributions of each feature, thereby illustrating how specific conventional radiomics, habitat radiomics, and clinicopathologic variables influenced the final prediction.

3.5. Features Explanation

Table 9-1 Biological Interpretation of Selected Radiomics Features Based on IBSI Definitions

| Feature | IBSI Category | Biological Interpretation |
| --- | --- | --- |
| wavelet-HLH_glcm  _Correlation_dwi | GLCM | Reflects linear dependency of gray levels; higher values indicate more organized microstructure and potentially increased cellular alignment in diffusion-restricted tissue. |
| wavelet-HHH_firstorder  _Mean_T2_habitat3 | First-order | Represents average signal intensity; in T2WI high metabolism habitats, higher values suggest increased rich blood supply and tissue breakdown. |
| log-sigma-3-0-mm-3D_  firstorder_Range_dyn_habitat3 | First-order | Measures intensity dispersion; high values indicate strong heterogeneity, often associated with necrosis or mixed vascularity. |
| log-sigma-4-0-mm-3D_glszm_  SizeZoneNonUniformityNormalized_T2WI | GLSZM | Quantifies variability of zone sizes; higher values reflect heterogeneous structural organization and irregular tumor architecture. |
| log-sigma-4-0-mm-3D  _glszm_ZoneEntropy_dwi | GLSZM | Indicates randomness of zone distribution; elevated entropy corresponds to complex microenvironment and high tumor heterogeneity. |
| log-sigma-4-0-mm-3D_glrlm_  ShortRunHighGrayLevelEmphasis_dwi | GLRLM | Emphasizes short runs of high intensity; associated with densely packed, highly cellular tumor regions. |
| wavelet-HHL_firstorder  _Uniformity_dyn_habitat1 | First-order | Measures homogeneity; higher values indicate uniform tissue composition, often corresponding to viable tumor regions. |
| wavelet-HLH_glcm_  Autocorrelation_T2WI | GLCM | Reflects repeating texture patterns; higher values suggest structured tissue organization such as fibrosis or stromal alignment. |
| wavelet-LLH_glszm_  GrayLevelNonUniformityNormalized_T2 | GLSZM | Measures variability of gray levels; higher values indicate heterogeneous tissue composition. |
| log-sigma-4-0-mm-3D  _firstorder_Maximum_dwi_habitat2 | First-order | Captures peak signal intensity; high values may reflect highly cellular or diffusion-restricted tumor subregions. |
| wavelet-HLH_glszm  _HighGrayLevelZoneEmphasis_dwi | GLSZM | Highlights large high-intensity zones; associated with dense cellular clusters or hypercellular tumor regions. |
| wavelet-LHL_gldm  _DependenceEntropy_dwi | GLDM | Measures complexity of voxel dependencies; higher values indicate heterogeneous microenvironment and complex cellular interactions. |
| original_firstorder  _InterquartileRange_dyn_habitat1 | First-order | Reflects dispersion of middle intensities; increased values indicate moderate heterogeneity in viable tumor tissue. |
| wavelet-LLH_glcm  _Correlation_dwi | GLCM | Measures spatial dependency; higher values suggest more homogeneous and organized diffusion patterns. |
| wavelet-HLH_glcm  _Autocorrelation_dyn | GLCM | Indicates repetitive texture structure; linked to organized vascular or stromal patterns. |
| wavelet-HLH_glszm_  LowGrayLevelZoneEmphasis  _dwi_habitat3 | GLSZM | Emphasizes low-intensity zones; associated with necrotic or cystic tumor regions. |
| wavelet-HLL_glszm_  SmallAreaLowGrayLevelEmphasis  _T2WI_habitat2 | GLSZM | Highlights small low-intensity areas; may correspond to micro-necrosis or early degenerative changes. |
| wavelet-HLL  _glcm_Correlation_T2WI | GLCM | Reflects spatial gray-level similarity; higher values indicate structured tissue organization. |
| wavelet-HLL_glszm  _SmallAreaEmphasis_T2WI | GLSZM | Captures fine-scale heterogeneity; associated with microstructural complexity such as capillary proliferation. |
| wavelet-HHL_glszm  _GrayLevelNonUniformity_T2WI | GLSZM | Measures variability of gray levels; higher values indicate heterogeneous signal intensity distribution. |
| wavelet-HHL_  firstorder_Kurtosis_T2WI | First-order | Reflects peakedness of intensity distribution; higher values suggest homogeneous cellular populations, lower values indicate heterogeneity. |
| wavelet-LHL_firstorder  _10Percentile_dyn_habitat1 | First-order | Represents low-intensity threshold; lower values may indicate hypoxic or poorly perfused regions. |
| log-sigma-3-0-mm-3D_glrlm  _ShortRunHighGrayLevelEmphasis_T2WI | GLRLM | Indicates short, high intensity runs; reflects fine, dense tissue structures. |
| wavelet-HLL_glszm_  SizeZoneNonUniformityNormalized  _T2WI_habitat1 | GLSZM | Reflects variability in zone size; higher values indicate heterogeneous structural organization. |
| original_shape_Elongation_T2WI | Shape | Describes tumor geometry; higher elongation suggests infiltrative growth patterns. |
| log-sigma-3-0-mm-3D_glszm_  SmallAreaHighGrayLevelEmphasis_DWI | GLSZM | Captures small, high-intensity zones; associated with microstructural heterogeneity and dense tumor clusters. |
| wavelet-LHL_glszm_  GrayLevelNonUniformity_T2WI_habitat2 | GLSZM | Indicates intensity variability; higher values reflect heterogeneous tissue composition. |
| original_firstorder_Variance_T2WI | First-order | Measures overall intensity variance; higher values correspond to increased heterogeneity and mixed tissue components. |
